# A developmental perspective on appearance-related social media use, body dissatisfaction, and competitive anxiety among Chinese adolescent athletes: a three-wave longitudinal study

**DOI:** 10.3389/fpsyg.2026.1805798

**Published:** 2026-04-16

**Authors:** Weimin Zhang, Rongxuan Duan, Haolan Li, Yu Fang, Weilun Zhao, Fang Wan, Jiangtao Fan

**Affiliations:** 1 College of Education, Nanchang Vocational University, Nanchang, Jiangxi, China; 2 Anhui Lvhai Vocational College of Business, Hefei, Anhui, China; 3 Department of Public Culture, Nanchang Health Vocational And Technical College, Nanchang, Jiangxi, China; 4 Taiji Culture College, Handan University, Handan, Hebei, China

**Keywords:** adolescent development, age differences, appearance-related social media use, body dissatisfaction, competitive anxiety, developmental differences

## Abstract

**Introduction:**

Digital media engagement centered on appearance has become ubiquitous among adolescents, yet its developmental trajectory and age-specific psychological impacts on young athletes remain poorly understood. This study investigates age-related differences in the longitudinal relationships between appearance-related social media use (ARSME), body dissatisfaction (BD), and competitive anxiety (CA) among Chinese adolescent athletes (aged 14–18) across aesthetic/weight-class and non-leanness sports. The developmental psychopathology framework was adopted because adolescence encompasses distinct developmental phases characterized by differential sensitivity to social-evaluative environmental influences, making a developmental lens essential for understanding age-specific vulnerability to appearance-focused digital content.

**Methods:**

A 12-month, three-wave longitudinal study was conducted with 356 Chinese adolescent athletes (184 males, 172 females; 14–18 years). Participants were categorized into younger (14–16 years, *n* = 200) and older (17–18 years, *n* = 156) groups. Cross-lagged panel modeling (CLPM), random intercept CLPM (RI-CLPM), and multi-group analyses were employed, controlling for sport type (aesthetic/weight-class vs. other sports).

**Results:**

Younger adolescents exhibited significantly higher levels of ARSME (*d* = 0.26–0.37), body dissatisfaction (*d* = 0.29–0.45), and competitive anxiety (*d* = 0.24–0.30). Age moderated the ARSME → body dissatisfaction pathway (Δχ^2^ = 5.28, *p* = 0.022), with stronger effects in younger (*β* = 0.22) than older (*β* = 0.13) athletes. Body dissatisfaction mediated 61% of the ARSME–competitive anxiety relationship in younger versus 30% in older adolescents. A supplementary sensitivity analysis decomposing ARSME into behavioral engagement and psychological investment components revealed that psychological investment showed a marginally significant prospective effect on body dissatisfaction (*β* = 0.08, *p* = 0.066), while behavioral screen time did not, providing preliminary support for cognitive-processing-focused interventions. Effects remained significant after controlling for sport type.

**Conclusion:**

Younger adolescent athletes (14–16 years) represent a critical period of heightened vulnerability. Prevention-focused interventions for younger adolescents should prioritize cognitive reappraisal training, metacognitive awareness skills, and functionality-focused body appreciation rather than mere screen time reduction. For older adolescents (17–18 years), interventions targeting performance-based social comparison and authentic self-presentation are additionally warranted.

## Introduction

The proliferation of social media has fundamentally altered the social landscape of adolescence. Recent surveys indicate that 95% of adolescents have access to smartphones and report near-constant online connectivity (Anderson and Jiang, 2018; Rideout, 2015). These digital platforms are characterized by content emphasizing physical appearance, idealized self-presentation, and appearance-based social comparisons (Fardouly and Vartanian, 2016; Perloff, 2014). For adolescent athletes, this digital environment presents unique developmental challenges: the functional athletic body shaped through training may conflict with culturally idealized appearance standards promoted on social media, potentially exacerbating body image concerns and undermining psychological readiness for competition (Kong and Harris, 2015; Varnes et al., 2013).

Despite substantial research linking social media use to body dissatisfaction in general adolescent populations (Holland and Tiggemann, 2016; Saiphoo and Vahedi, 2019; Mingoia et al., 2017), critical gaps remain in understanding how these relationships operate across developmental periods. Adolescence is not a monolithic stage; rather, it encompasses distinct developmental phases characterized by differential vulnerability to environmental influences (Steinberg et al., 2009; Dahl et al., 2018). The present study addresses this gap by adopting a developmental psychopathology perspective (Cicchetti and Rogosch, 2002; Sroufe and Rutter, 1984) to examine age-related differences in the prospective relationships between appearance-related social media engagement (ARSME), body dissatisfaction, and competitive anxiety among Chinese adolescent athletes.

### Developmental framework: why age matters

Developmental theory provides compelling reasons to expect age-related differences in vulnerability to appearance-focused digital content. First, Erikson (1968) psychosocial theory posits that adolescents navigate the central crisis of identity versus role confusion, with early-to-mid adolescence (approximately 14–16 years) representing a particularly sensitive period for self-concept formation (Kroger et al., 2010; Meeus, 2011). During this phase, individuals are especially attuned to external feedback and social evaluation, making them potentially more susceptible to appearance-related messaging on social media (Harter, 2015; Sebastian et al., 2010).

Second, the dual-system model (Steinberg, 2010) provides a precise account of developmental asymmetry in vulnerability to appearance-focused digital content. The 14–16 age period is characterized by a social–emotional system that is already highly active and acutely sensitive to peer evaluation, while the prefrontal cognitive control system remains incompletely developed. This imbalance renders younger adolescents simultaneously more reactive to appearance-related social stimuli and less equipped to regulate the resulting emotional responses—making them particularly susceptible to body image impacts from appearance-focused social media content. Neurodevelopmental research supports this framework, indicating that the prefrontal cortex—the brain region responsible for executive functions including impulse control, future orientation, and emotion regulation—continues to mature throughout adolescence (Casey et al., 2008; Blakemore and Choudhury, 2006). Critically, this maturation follows a nonlinear trajectory, with significant structural and functional changes occurring around age 16–17 that mark a qualitative shift in cognitive control capacities (Luna et al., 2010; Giedd et al., 1999). This neurodevelopmental inflection point provides theoretical justification for distinguishing early-to-mid adolescence (14–16 years) from late adolescence (17–18 years), as the latter group has typically crossed this maturational threshold and possesses more developed cognitive resources for critically evaluating idealized digital content and regulating emotional responses to appearance-based social comparisons (Crone and Dahl, 2012; Albert and Steinberg, 2011).

Third, pubertal development and associated bodily changes peak during early-to-mid adolescence (Susman and Dorn, 2012; Mendle et al., 2007). This period of rapid physical transformation heightens body awareness and may increase sensitivity to appearance-related feedback (Graber et al., 2010). When combined with exposure to idealized body images on social media, this developmental context may create a “perfect storm” for body image disturbance in younger adolescents (Rodgers et al., 2020; McLean et al., 2016). Furthermore, the temporal asynchrony between pubertal maturation and cognitive development, characterized by physical changes preceding the development of regulatory capacities, may render younger adolescents particularly vulnerable (Dahl, 2004).

Finally, older adolescents (17–18 years) may benefit from accumulated experiences that foster resilience. They have typically developed more stable self-concepts, refined coping strategies, and greater media literacy skills that enable more critical engagement with digital content (Livingstone, 2014; Valkenburg and Peter, 2013). Research on identity development suggests that by late adolescence, most individuals have achieved greater identity coherence, providing a buffer against external appearance pressures (Klimstra et al., 2010).

### ARSME and body dissatisfaction: a developmental perspective

Appearance-related social media engagement encompasses both behavioral involvement (time spent on platforms) and psychological investment (concern about how one’s appearance is perceived online) (Choukas-Bradley et al., 2020; Nesi and Prinstein, 2015). This dual-component conceptualization is critical because it captures not only the quantity of exposure but also the quality of cognitive processing, reflecting the degree to which individuals internalize and ruminate on appearance-related online feedback (Burnell et al., 2020; Tiggemann and Slater, 2014). Cross-sectional research has established associations between appearance-focused social media behaviors and body dissatisfaction in general adolescent samples (Saiphoo and Vahedi, 2019; Grabe et al., 2008). However, longitudinal studies examining these processes in athlete populations remain scarce (Gattario and Frisén, 2019).

Building on objectification theory (Fredrickson and Roberts, 1997), which posits that repeated exposure to objectifying environments promotes self-objectification and body surveillance, we propose that ARSME functions as a developmentally sensitive risk factor. The tripartite influence model (Thompson et al., 1999; Keery et al., 2004) further suggests that media, peers, and parents transmit appearance ideals that promote body dissatisfaction through social comparison and internalization processes. Younger adolescents, with their still-developing identity and limited regulatory capacities, may be particularly vulnerable to internalizing the objectifying messages prevalent on social media platforms (Slater and Tiggemann, 2015; Tiggemann and Miller, 2010).

### Body dissatisfaction and competitive anxiety: a universal mechanism?

Competitive anxiety, characterized by cognitive worry, somatic symptoms, and diminished confidence prior to competition, is a reliable predictor of impaired athletic performance, reduced enjoyment, and premature sport dropout (Smith et al., 2006; Grossbard et al., 2009). The multidimensional anxiety theory (Martens et al., 1990) and processing efficiency theory (Eysenck and Calvo, 1992; Eysenck et al., 2007) provide frameworks for understanding how cognitive concerns, particularly those related to body image, may interfere with athletic performance.

Self-determination theory (Deci and Ryan, 2000; Ryan and Deci, 2024) suggests that body dissatisfaction undermines the fundamental psychological need for competence. Athletes preoccupied with appearance-related concerns may experience diminished confidence in their physical capabilities, thereby increasing vulnerability to pre-competition anxiety. This theoretical link has received preliminary support in cross-sectional research with female athletes (Francisco et al., 2013; De Bruin et al., 2007).

Importantly, while the pathways leading to body dissatisfaction may vary developmentally, we hypothesize that once body dissatisfaction is established, its downstream effects on competitive anxiety operate through relatively universal mechanisms that transcend developmental stage. Body dissatisfaction, regardless of its origins, represents a fundamental disruption to the athlete’s relationship with their physical self, serving as the very instrument of their competitive performance. This disruption may activate anxiety through common pathways: heightened self-consciousness during competition (Leary, 1992), attentional interference from appearance-monitoring (Tiggemann and Slater, 2001), and undermined confidence in physical capabilities (Hausenblas and Fallon, 2006). These mechanisms reflect basic psychological processes that are unlikely to differ substantially between younger and older adolescents.

### The Chinese cultural context

The present study focuses on Chinese adolescent athletes, a population that merits specific attention. China has witnessed rapid social media proliferation, with platforms such as WeChat, Weibo, and Douyin (TikTok) reaching near-universal adoption among youth (Cnnic, 2018). Traditional Chinese beauty ideals emphasizing fair skin, slender physique, and delicate features (Jung and Lee, 2006; Luo, 2013) may conflict with athletic body types, potentially intensifying body image concerns among Chinese adolescent athletes. Additionally, the cultural emphasis on “face” (mianzi) and social harmony may heighten sensitivity to perceived online evaluations of one’s appearance (Qi, 2011). Despite these cultural specificities, the fundamental developmental processes underlying vulnerability to appearance-focused media are likely to share universal features while manifesting within culturally specific contexts (Chen and French, 2008).

### The present study

This 12-month, three-wave longitudinal study examines the developmental dynamics of ARSME, body dissatisfaction, and competitive anxiety among Chinese adolescent athletes. We extend previous research by: (1) adopting a developmental psychopathology framework to examine age as a moderator of prospective relationships; (2) comparing younger (14–16 years) and older (17–18 years) adolescent athletes based on neurodevelopmental theory; (3) controlling for sport type given differential appearance pressures across athletic disciplines; and (4) utilizing both traditional cross-lagged panel models (CLPM) (Selig and Little, 2012) and random intercept cross-lagged panel models (RI-CLPM) (Hamaker et al., 2015) to distinguish between-person and within-person effects.

We test the following hypotheses:

H1: Younger adolescent athletes will exhibit higher levels of ARSME, body dissatisfaction, and competitive anxiety compared to older adolescents.

H2: ARSME will prospectively predict increases in body dissatisfaction, with stronger effects among younger adolescents (age moderation).

H3: Body dissatisfaction will prospectively predict increases in competitive anxiety, with similar effect sizes across age groups (no age moderation).

H4: Body dissatisfaction will mediate the ARSME–competitive anxiety relationship, with stronger mediation effects among younger adolescents.

## Methods

### Participants and recruitment

Participants were 356 adolescent athletes (184 males, 172 females; M = 16.11 years, SD = 1.11, range = 14–18 years) recruited from four sports schools in Jiangxi Province, China. Based on developmental neuroscience literature identifying age 16–17 as a critical inflection point in prefrontal cortex maturation (Luna et al., 2010; Steinberg, 2017), participants were classified into a younger group (14–16 years; *n* = 200, 56.2%; M = 15.30 years, SD = 0.74) and an older group (17–18 years; *n* = 156, 43.8%; M = 17.15 years, SD = 0.41). This categorical approach facilitates multi-group structural equation modeling while being grounded in neurodevelopmental theory; supplementary analyses treating age as a continuous moderator are also reported (Little, 2013).

The sample represented 18 sport categories—gymnastics, diving, figure skating, wrestling, judo, weightlifting, boxing, taekwondo, wushu, track and field, swimming, basketball, volleyball, football (soccer), table tennis, badminton, rowing, and shooting—which were classified into two groups based on appearance-related pressures: (1) aesthetic and weight-class sports (*n* = 120; 33.7%), including gymnastics, diving, figure skating, wrestling, and judo; and (2) other sports (*n* = 236; 66.3%), including endurance and team/strength sports. This classification follows established frameworks distinguishing “leanness-focused” from “non-leanness-focused” sports (Kong and Harris, 2015; Sundgot-Borgen and Torstveit, 2004). Critically, the distribution of sport types did not differ significantly between age groups (χ^2^ = 0.43, *p* = 0.510), reducing concerns about confounding.

Participants competed primarily at the school and provincial levels (70.2 and 29.8%, respectively); none were competing at the national level at time of recruitment. Participants trained an average of 18.3 h per week (SD = 5.7) and had been involved in competitive sports for an average of 4.8 years (SD = 2.1). Of 410 athletes initially contacted, 398 agreed to participate (recruitment rate = 97.1%). Attrition between T1 and T3 was 10.6%, yielding 356 complete datasets. To assess potential attrition bias, the 42 non-completers were compared with the 356 completers on all baseline variables. Non-completers did not differ significantly from completers in age group distribution (χ^2^ = 0.84, *p* = 0.36), gender (χ^2^ = 1.12, *p* = 0.29), baseline ARSME (*t* = 1.03, *p* = 0.30), baseline body dissatisfaction (*t* = 0.87, *p* = 0.39), or baseline competitive anxiety (*t* = 1.21, *p* = 0.23), suggesting that attrition is unlikely to introduce systematic bias. Little’s MCAR test confirmed data were missing completely at random (χ^2^ = 142.35, df = 156, *p* = 0.783), supporting the use of full information maximum likelihood estimation (Enders and Bandalos, 2001).

*Sample Size Justification. A priori* power analysis was conducted using G*Power 3.1 (Faul et al., 2009). Based on previous longitudinal research examining social media–body image associations (*r* = 0.23) (Saiphoo and Vahedi, 2019), we estimated a small-to-medium effect size (f^2^ = 0.05). For cross-lagged panel models with 9 observed variables, *α* = 0.05, and powe*r* = 0.80, a minimum sample of 324 participants was required (Wolf et al., 2013). Our final sample of 356 exceeded this threshold.

### Procedure

This study was approved by the Ethics Committee of Nanchang Vocational University (Approval No. NCVN-24RT-2403). Informed consent was obtained from all participants and their parents/guardians (for participants under 18 years of age) prior to data collection. Data were collected at three time points, each separated by six months: Time 1 (T1; March 2024), Time 2 (T2; September 2024), and Time 3 (T3; March 2025). The six-month intervals aligned with competitive seasons and provided adequate time to observe prospective effects while minimizing memory biases (Collins, 2006). Participants completed online questionnaires via a secure platform during scheduled group sessions, with each session taking approximately 20–25 min to complete. Trained research assistants provided standardized instructions, emphasizing voluntary participation, confidentiality, and the right to withdraw.

### Measures

#### Appearance-related social media engagement (ARSME)

ARSME was operationalized as a composite measure integrating two theoretically distinct components: (1) behavioral engagement, defined as daily social media time (0 = <30 min to 8 = > 7 h); and (2) psychological investment, measured by the Appearance-Related Social Media Consciousness (ASMC) (Choukas-Bradley et al., 2020), a 13-item scale assessing cognitive preoccupation with how one’s appearance is perceived online (e.g., “I think about what I look like in photos on social media”; *α* = 0.88–0.90 across waves). To construct the ARSME composite, both sub-components were first z-standardized to a common metric before averaging, because behavioral engagement (scored 0–8) and psychological investment (Likert 1–5) carry different scoring ranges; this standardization ensures neither sub-component disproportionately dominates composite variance. We note that combining behavior and psychological investment into a single composite may mask unique effects of each component; a sensitivity analysis decomposing these sub-components is reported in the Supplementary analyses section. This composite approach captures both the quantity and quality of appearance-focused digital engagement, consistent with theoretical models emphasizing that psychological investment in online feedback, rather than mere exposure, drives negative outcomes (Valkenburg and Peter, 2013; Burnell et al., 2020).

#### Body dissatisfaction

Body dissatisfaction was assessed using the Negative Physical Self Scale (NPSS) (Chen et al., 2006), a 48-item measure validated for Chinese adolescents. The scale assesses dissatisfaction across five domains: obesity, underweight, height, facial features, and overall appearance. Items were rated from 0 (strongly disagree) to 4 (strongly agree), with higher mean scores indicating greater dissatisfaction (*α* = 0.94–0.95 across waves). Measurement invariance testing confirmed scalar invariance across time (ΔCFI ≤ 0.008) and age groups (ΔCFI ≤ 0.009) (Putnick and Bornstein, 2016).

#### Competitive anxiety

Pre-competition anxiety was measured using the Chinese version of the Competitive State Anxiety Inventory-2 (CSAI-2) (Martens et al., 1990), adapted and normed for Chinese athletes by Zhu (1994). This 27-item scale assesses three dimensions: cognitive anxiety, somatic anxiety, and self-confidence. Items were rated from 1 (not at all) to 4 (very much so), with higher mean scores indicating greater anxiety (α = 0.91–0.93 across waves). The Chinese CSAI-2 has demonstrated adequate internal consistency (α = 0.68–0.72) and good concurrent validity with trait anxiety measures in Chinese athlete samples (Zhu, 1994). The higher α values observed in the current sample (relative to *α* = 0.68–0.72 in the original Chinese validation) (Zhu, 1994) likely reflect the homogeneous nature of our specialized sports school sample, wherein reduced between-participant variance on anxiety-related items can elevate internal consistency estimates.

#### Sport type

Coded as a binary covariate: aesthetic/weight-class sports (1) versus other sports (0). This variable was included as a covariate in all models to control for differential appearance pressures across athletic disciplines (Sundgot-Borgen and Torstveit, 2004).

### Data analysis

Analyses proceeded in six stages. First, we conducted preliminary analyses verifying sport type distribution across age groups and examined whether age-group differences remained significant after controlling for sport type using ANCOVA. Second, we estimated a CLPM including ARSME, body dissatisfaction, and competitive anxiety at all three time points with sport type as a covariate. The CLPM comprises three types of paths: (a) autoregressive paths, capturing stability of each construct across waves; (b) cross-lagged paths, representing prospective prediction of one construct by another after controlling for prior levels; and (c) synchronous (contemporaneous) correlations among residuals at each wave. All models were estimated using the Maximum Likelihood with Robust standard errors (MLR) estimator, given its robustness to non-normality. Missing data were handled using full information maximum likelihood (FIML) throughout. Third, we conducted multi-group CLPM analyses with age group as the grouping variable and used chi-square difference tests to evaluate age moderation of specific paths (Satorra and Bentler, 2001). Prior to multi-group comparisons, measurement invariance of all indicators across age groups was tested via a sequence of configural, metric, and scalar models; scalar invariance was confirmed (ΔCFI ≤ 0.009 across all steps), supporting meaningful cross-group comparisons. Fourth, we estimated RI-CLPM to decompose variance into stable between-person differences and within-person fluctuations (Hamaker et al., 2015). Fifth, as a supplementary analysis, we tested age as a continuous moderator using latent moderated structural equations (LMS) (Klein and Moosbrugger, 2000). The LMS approach estimates latent interaction effects by treating the joint distribution of predictor and moderator as a mixture distribution, thereby avoiding normality assumptions for the interaction term. Sixth, we conducted a component-level sensitivity analysis in which behavioral engagement and psychological investment were entered as separate z-standardized predictors in the CLPM, replacing the ARSME composite, to test whether the prospective effects on body dissatisfaction are primarily driven by behavioral exposure or by cognitive preoccupation.

Model fit was evaluated using standard criteria: CFI ≥ 0.95, TLI ≥ 0.95, RMSEA ≤ 0.06, SRMR ≤ 0.08 (Hu and Bentler, 1999). Model fit comparisons were supplemented with AIC and BIC where applicable. Mediation effects were tested using the product of coefficients method with bias-corrected bootstrap confidence intervals (10,000 resamples) (MacKinnon et al., 2002). All analyses were conducted using R 4.3.1 with lavaan (Rosseel, 2012) and semTools packages.

## Results

### Preliminary analyses

#### Sport type distribution

The distribution of sport types did not differ significantly between younger (32.0% aesthetic/weight-class) and older (35.9% aesthetic/weight-class) adolescents (χ2 = 0.43, *p* = 0.510), indicating that any observed age differences are unlikely to be confounded by sport type composition.

Descriptive Statistics. Table 1 presents descriptive statistics and zero-order correlations for all study variables. Across time points, ARSME was positively correlated with body dissatisfaction (*r* = 0.26–0.41, all ps < 0.001) and competitive anxiety (*r* = 0.18–0.25, ps < 0.01–0.001). Body dissatisfaction and competitive anxiety showed consistent positive associations (*r* = 0.24–0.38, all ps < 0.001). Test–retest reliability indicated moderate-to-high stability for all constructs (rs = 0.60–0.71). Univariate normality assumptions were satisfied (|skewness| < 2.0, |kurtosis| < 7.0) (Kline, 2023). Specifically, skewness ranged from −0.37 to 0.33 and kurtosis from −0.70 to 0.32 across all study variables at all time points.

**Table 1:** Descriptive statistics and correlations.

Variable	M	SD	1	2	3	4	5	6	7	8	9
1. ARSME T1	2.90	0.79	—								
2. ARSME T2	2.84	0.86	0.68***	—							
3. ARSME T3	2.93	0.82	0.62***	0.71***	—						
4. BD T1	2.45	0.79	0.34***	0.28***	0.26***	—					
5. BD T2	2.52	0.85	0.31***	0.38***	0.32***	0.66***	—				
6. BD T3	2.56	0.84	0.29***	0.34***	0.41***	0.61***	0.69***	—			
7. CA T1	2.38	0.74	0.22***	0.19**	0.18**	0.31***	0.27***	0.24***	—		
8. CA T2	2.41	0.77	0.20**	0.24***	0.21***	0.29***	0.35***	0.30***	0.64***	—	
9. CA T3	2.46	0.76	0.18**	0.22***	0.25***	0.27***	0.31***	0.38***	0.60***	0.67***	—

*N* = 356. ARSME, appearance-related social media engagement; BD, body dissatisfaction; CA, competitive anxiety. **p < 0.01; ***p < 0.001.

#### Common method bias

Harman’s single-factor test indicated that the first unrotated factor explained 29.4% of the variance, below the 50% threshold (Podsakoff et al., 2003). A common latent factor model revealed that the unmeasured factor explained 18.6% of variance, suggesting minimal influence of common method bias.

### Age-related differences in study variables (H1)

**Figure 1:**
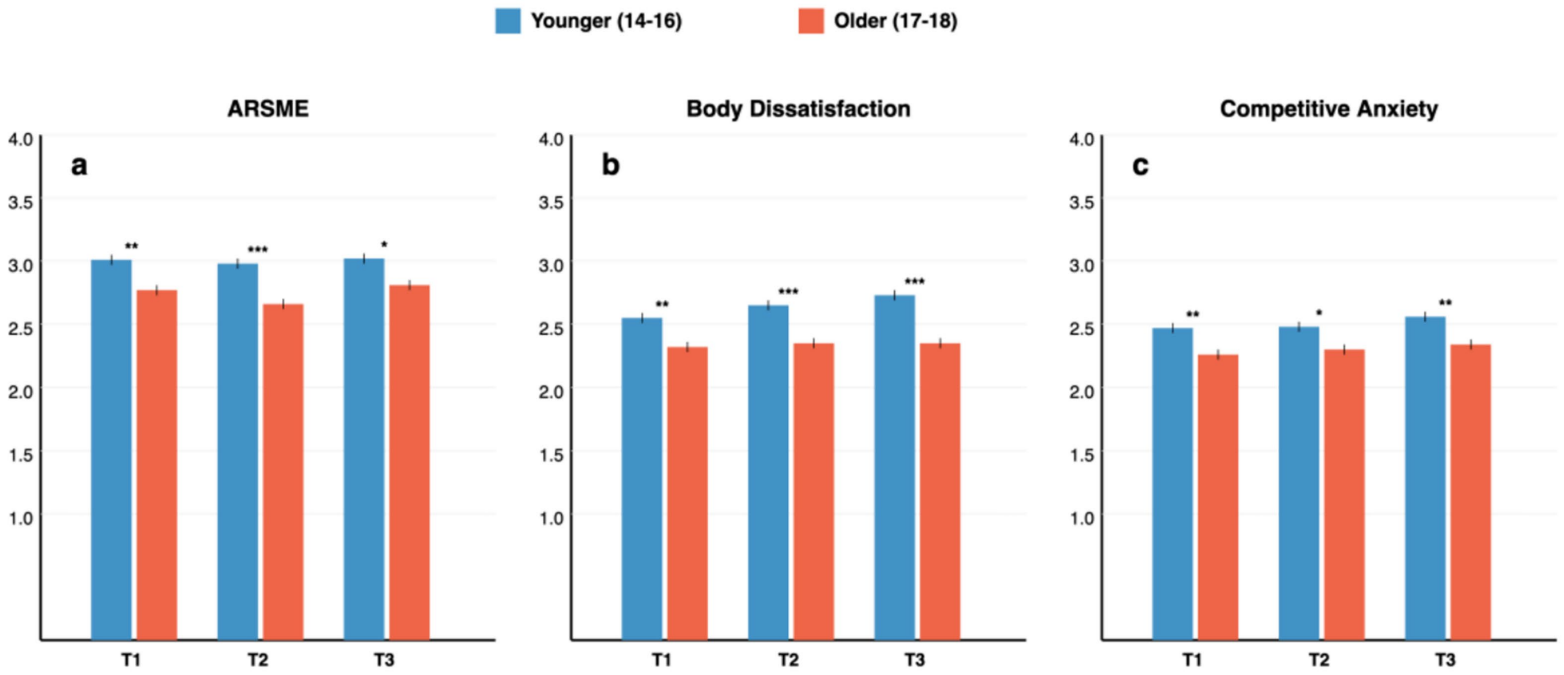
Age group comparisons on ARSME, body dissatisfaction, and competitive anxiety across time points. Error bars = SE. Differences remained significant after controlling for sport type. *p < 0.05; **p < 0.01; ***p < 0.001.

Table 2 presents comparisons between younger (14–16 years) and older (17–18 years) adolescent athletes. Supporting Hypothesis 1, younger adolescents exhibited significantly higher levels across all study variables and time points. Critically, these differences remained significant after controlling for sport type in ANCOVA models (all ps < 0.05), confirming that age effects are independent of sport type distribution. Figure 1 visualizes these age-group differences across the three time points.

**Table 2:** Age-related differences in study variables.

Variable	Younger (14–16) *n* = 200 M (SD)	Older (17–18) *n* = 156 M (SD)	*t*	*p*	*d*
ARSME T1	3.01 (0.77)	2.77 (0.80)	2.89	0.004	0.31
ARSME T2	2.98 (0.88)	2.66 (0.82)	3.45	<0.001	0.37
ARSME T3	3.02 (0.87)	2.81 (0.74)	2.39	0.017	0.26
BD T1	2.55 (0.86)	2.32 (0.66)	2.71	0.007	0.29
BD T2	2.65 (0.84)	2.35 (0.82)	3.40	<0.001	0.36
BD T3	2.73 (0.88)	2.35 (0.74)	4.22	<0.001	0.45
CA T1	2.47 (0.76)	2.26 (0.70)	2.61	0.009	0.28
CA T2	2.48 (0.77)	2.30 (0.75)	2.20	0.028	0.24
CA T3	2.56 (0.79)	2.34 (0.70)	2.83	0.004	0.30

Sport type distribution did not differ between groups (χ^2^ = 0.43, *p* = 0.510). All differences remained significant after controlling for sport type (ANCOVA, ps < 0.05).

#### ARSME

Younger adolescents reported significantly higher ARSME at T1 (M = 3.01, SD = 0.77) than older adolescents (M = 2.77, SD = 0.80; *t* = 2.89, *p* = 0.004, *d* = 0.31). This pattern persisted at T2 (*d* = 0.37, *p* < 0.001) and T3 (*d* = 0.26, *p* = 0.017). The consistent age differences suggest that appearance-focused digital engagement declines developmentally across late adolescence.

#### Body dissatisfaction

**Figure 2:**
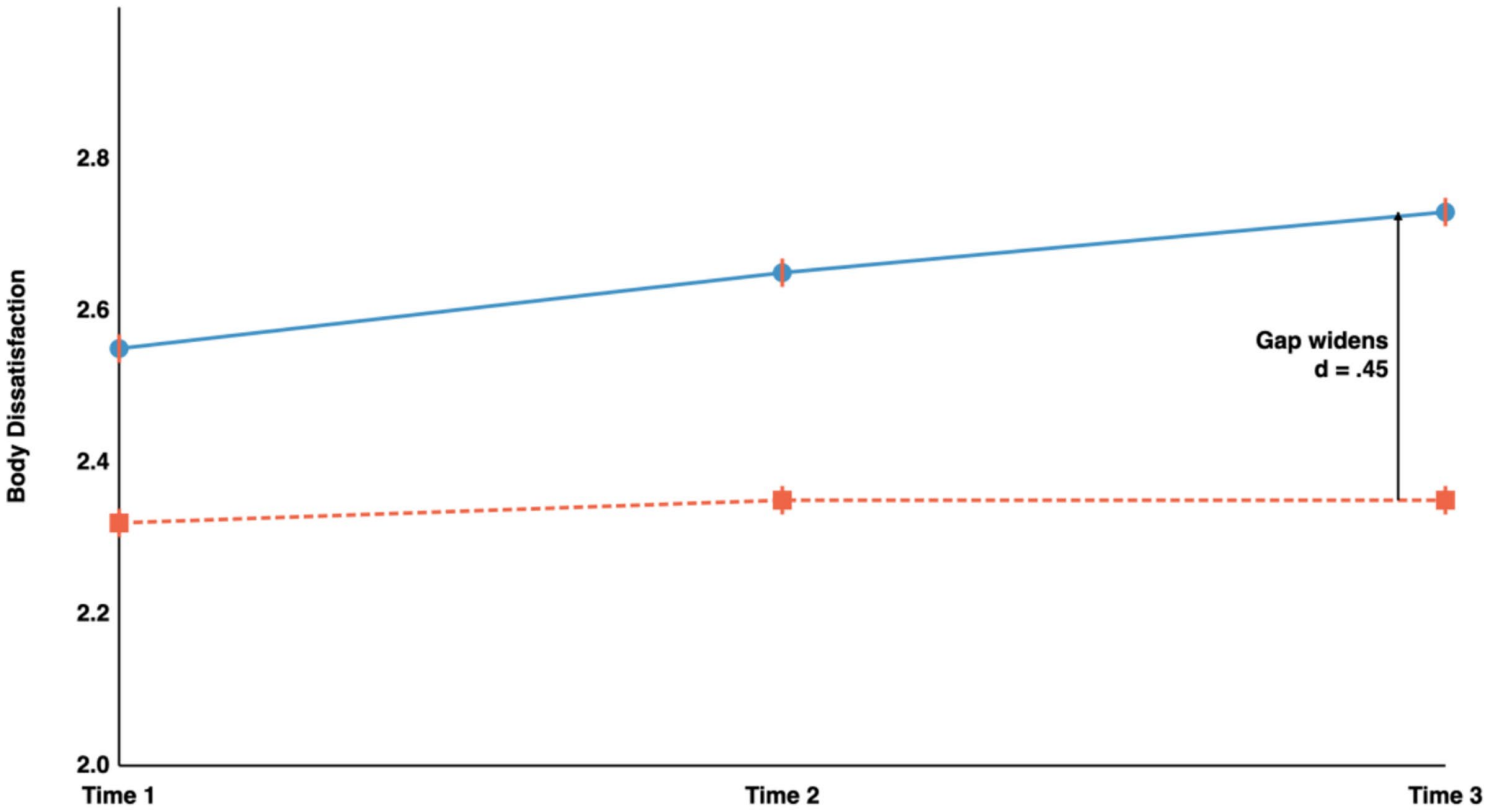
Developmental trajectories of body dissatisfaction. Age gap widened over time (*d* = 0.29 → 0.45), indicating diverging trajectories. Error bars = SE.

Younger adolescents exhibited significantly higher body dissatisfaction at all time points (T1: *d* = 0.29, a small effect, *p* = 0.007; T2: *d* = 0.36, a small-to-medium effect, *p* < 0.001; T3: *d* = 0.45, a medium effect, *p* < 0.001). Notably, the age gap widened over time (d increased from 0.29 to 0.45), as illustrated in Figure 2, indicating divergent developmental trajectories whereby younger adolescents showed relative increases in body dissatisfaction while older adolescents remained stable.

#### Competitive anxiety

Similarly, younger adolescents reported higher competitive anxiety at T1 (M = 2.47, SD = 0.76) compared to older adolescents (M = 2.26, SD = 0.70; *t* = 2.61, *p* = 0.009, *d* = 0.28). Age differences remained significant across subsequent waves (T2: *d* = 0.24, *p* = 0.028; T3: *d* = 0.30, *p* = 0.004).

### Cross-lagged panel model (H2, H3)

**Figure 3:**
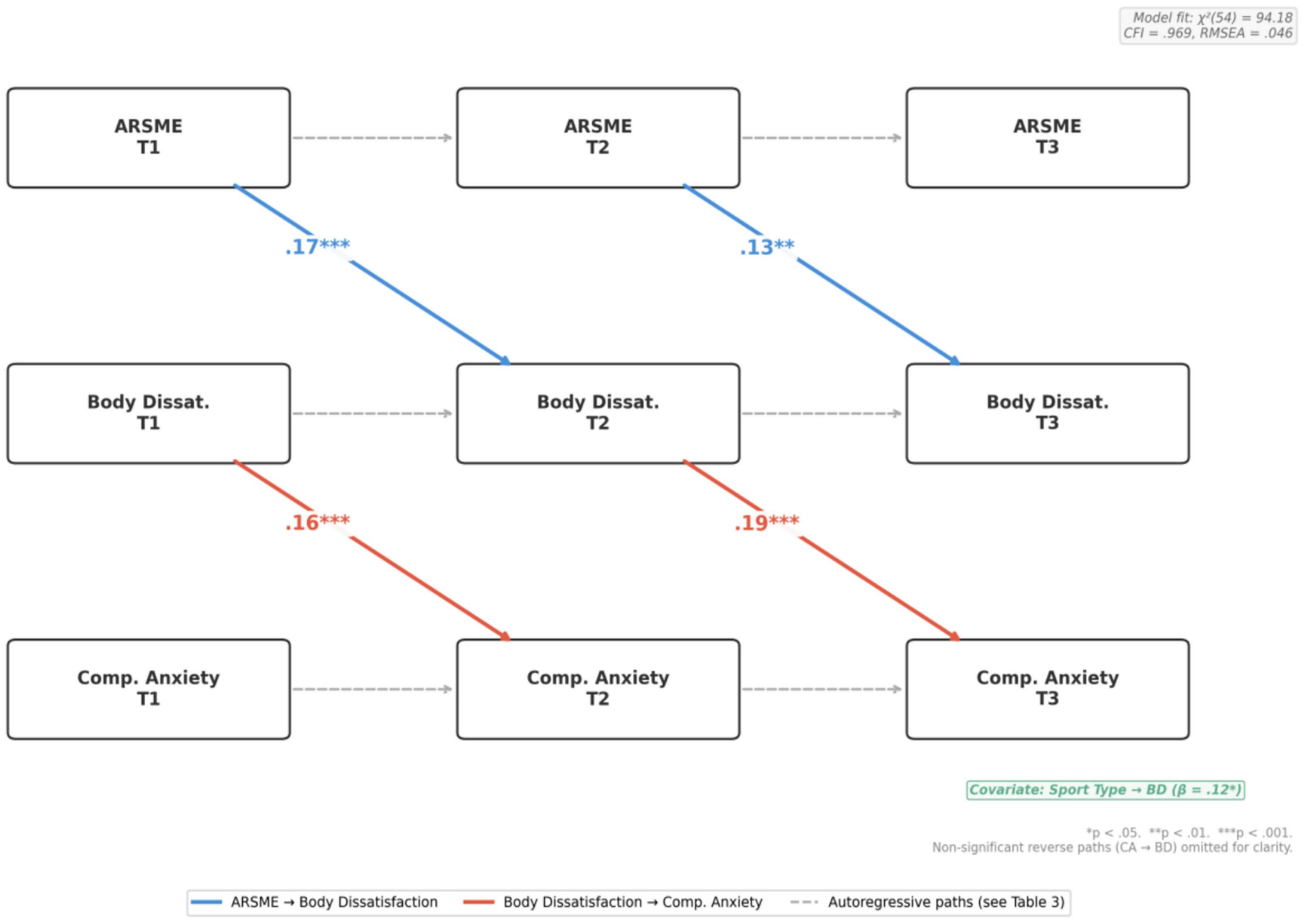
Cross-lagged panel model (controlling for sport type). All path coefficients shown are standardized (*β*) and have been estimated controlling for sport type as a covariate. Only key cross-lagged path coefficients are displayed for clarity; full autoregressive and reverse-path coefficients are reported in Table 3. Blue arrows: ARSME → BD; Red arrows: BD → CA; dashed gray: autoregressive paths; dotted: non-significant reverse paths (omitted). Model fit: χ^2^(54) = 94.18, CFI = 0.969, RMSEA = 0.046.

The CLPM with sport type as a covariate demonstrated excellent fit: χ^2^(54) = 94.18, *p* < 0.001; CFI = 0.969, TLI = 0.954, RMSEA = 0.046, 90% CI [0.030, 0.061], SRM*R* = 0.041. Table 3 presents standardized path coefficients, and Figure 3 illustrates the complete model. Sport type showed significant associations with body dissatisfaction (*β* = 0.12, *p* = 0.018) but not competitive anxiety (*β* = 0.06, *p* = 0.24), confirming the importance of controlling for athletic discipline.

**Table 3:** CLPM path coefficients (controlling for sport type).

Path	*β*	SE	95% CI	*p*
Covariate: Sport type → BD T1	0.12	0.05	[0.02, 0.22]	0.018
Covariate: Sport type → CA T1	0.06	0.05	[−0.04, 0.16]	0.238
ARSME T1 → BD T2	0.17	0.04	[0.09, 0.25]	<0.001
ARSME T2 → BD T3	0.13	0.04	[0.05, 0.21]	0.003
BD T1 → CA T2	0.16	0.04	[0.08, 0.24]	<0.001
BD T2 → CA T3	0.19	0.04	[0.11, 0.27]	<0.001
CA T1 → BD T2 (reverse)	0.06	0.04	[−0.02, 0.14]	0.142
CA T2 → BD T3 (reverse)	0.04	0.04	[−0.04, 0.12]	0.286

Model fit: χ^2^(54) = 94.18, CFI = 0.969, TLI = 0.954, RMSEA = 0.046, SRM*R* = 0.041. Sport type: 1 = aesthetic/weight-class, 0 = other.

#### Autoregressive effects

All constructs showed moderate-to-strong stability: ARSME (*β*T1 → T2 = 0.68, βT2 → T3 = 0.71), body dissatisfaction (*β*T1 → T2 = 0.66, βT2 → T3 = 0.69), and competitive anxiety (*β*T1 → T2 = 0.64, βT2 → T3 = 0.67), all ps < 0.001.

#### Cross-lagged effects: ARSME → body dissatisfaction (H2)

ARSME at T1 significantly predicted higher body dissatisfaction at T2 (*β* = 0.17, SE = 0.04, *p* < 0.001, [0.09, 0.25]), and ARSME at T2 predicted body dissatisfaction at T3 (*β* = 0.13, SE = 0.04, *p* = 0.003, [0.05, 0.21]). These effects remained significant after controlling for sport type, supporting H2.

#### Cross-lagged effects: body dissatisfaction → competitive anxiety (H3)

Body dissatisfaction at T1 predicted higher competitive anxiety at T2 (*β* = 0.16, SE = 0.04, *p* < 0.001, [0.08, 0.24]), and body dissatisfaction at T2 predicted competitive anxiety at T3 (*β* = 0.19, SE = 0.04, *p* < 0.001, [0.11, 0.27]). Reverse paths (competitive anxiety → body dissatisfaction) were non-significant (βs = 0.04–0.06, ps > 0.14), supporting unidirectional effects consistent with H3.

### Age moderation of cross-lagged paths

Table 4 presents the results of multi-group CLPM testing age moderation, controlling for sport type within each group. The unconstrained model fit the data well: χ^2^(108) = 168.52, *p* < 0.001, CFI = 0.965, TLI = 0.948, RMSEA = 0.048, SRM*R* = 0.044.

**Table 4:** Multi-group CLPM: age moderation tests.

Path	Younger β	Older β	Δχ^2^	df	*p*
ARSME → BD (T1 → T2)	0.22***	0.13*	5.28	1	0.022
ARSME → BD (T2 → T3)	0.15**	0.10	1.21	1	0.271
BD → CA (T1 → T2)	0.18***	0.14**	0.98	1	0.322
BD → CA (T2 → T3)	0.21***	0.17**	0.45	1	0.502

Continuous age moderation (LMS) confirmed: Age × ARSME *β* = −0.09, *p* = 0.018; Age × BD *β* = −0.02, *p* = 0.58. *p < 0.05; **p < 0.01; ***p < 0.001.

#### ARSME → body dissatisfaction

**Figure 4:**
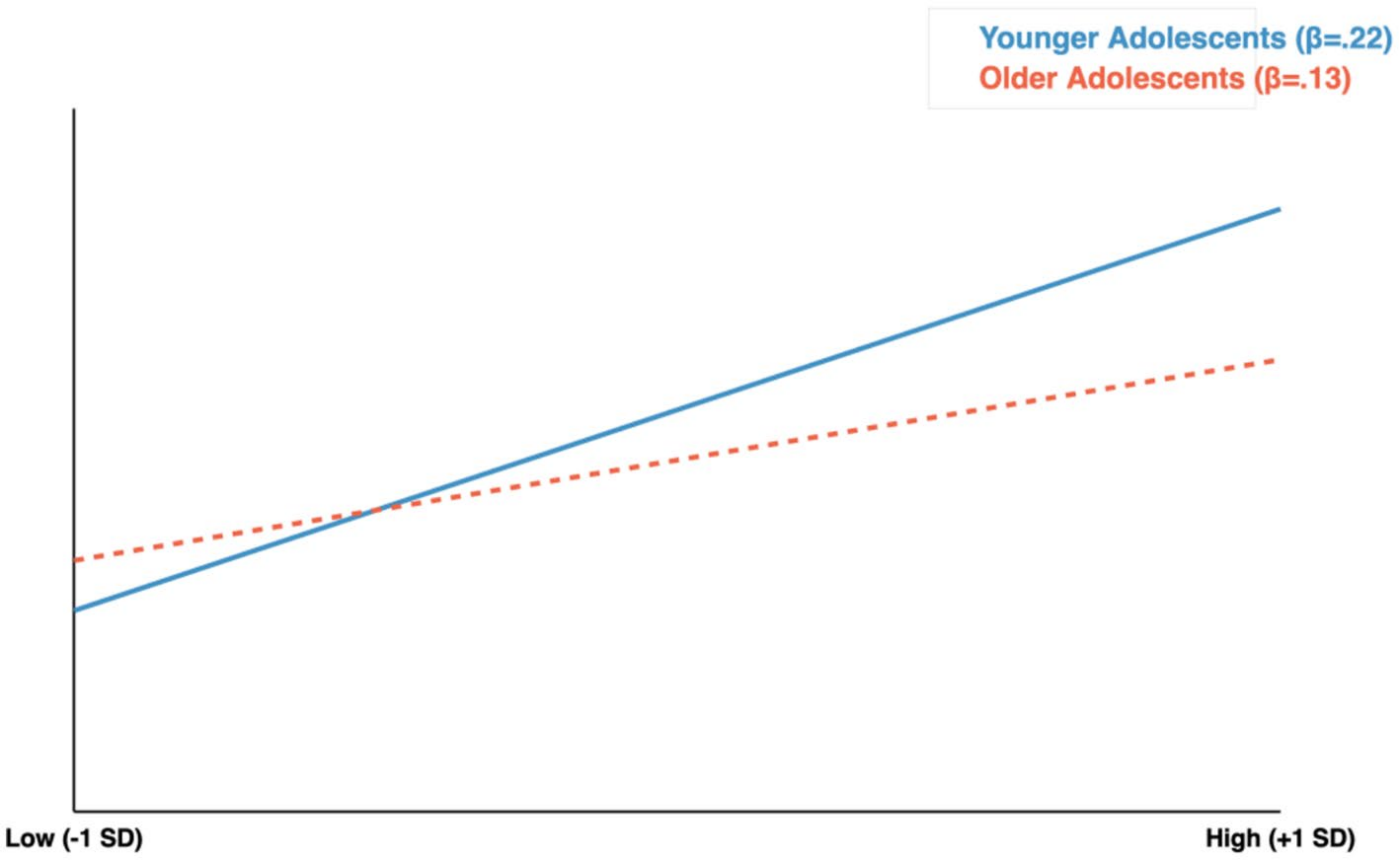
Age moderation of ARSME → body dissatisfaction. Effect stronger in younger (*β* = 0.22) than older (*β* = 0.13) adolescents. Age moderation: Δχ^2^ = 5.28; *p* = 0.022. Continuous analysis confirmed: *β* = −0.09; *p* = 0.018.

Age significantly moderated this path from T1 to T2 (Δχ^2^ = 5.28, df = 1, *p* = 0.022). As shown in Figure 4, the effect was significantly stronger in younger adolescents (*β* = 0.22, *p* < 0.001) than older adolescents (*β* = 0.13, *p* = 0.048). However, age moderation was not significant from T2 to T3 (Δχ^2^ = 1.21, *p* = 0.271), with effects becoming more similar across groups.

#### Body dissatisfaction → competitive anxiety

Consistent with H3, age did *not* moderate this path at either interval (T1–T2: Δχ^2^ = 0.98, *p* = 0.322; T2–T3: Δχ^2^ = 0.45, *p* = 0.502). The effect sizes were comparable across age groups (younger: *β* = 0.18–0.21; older: *β* = 0.14–0.17), supporting the hypothesis that once body dissatisfaction develops, its anxiety-provoking effects operate through relatively universal mechanisms regardless of developmental stage.

### Mediation analysis (H4)

**Figure 5:**
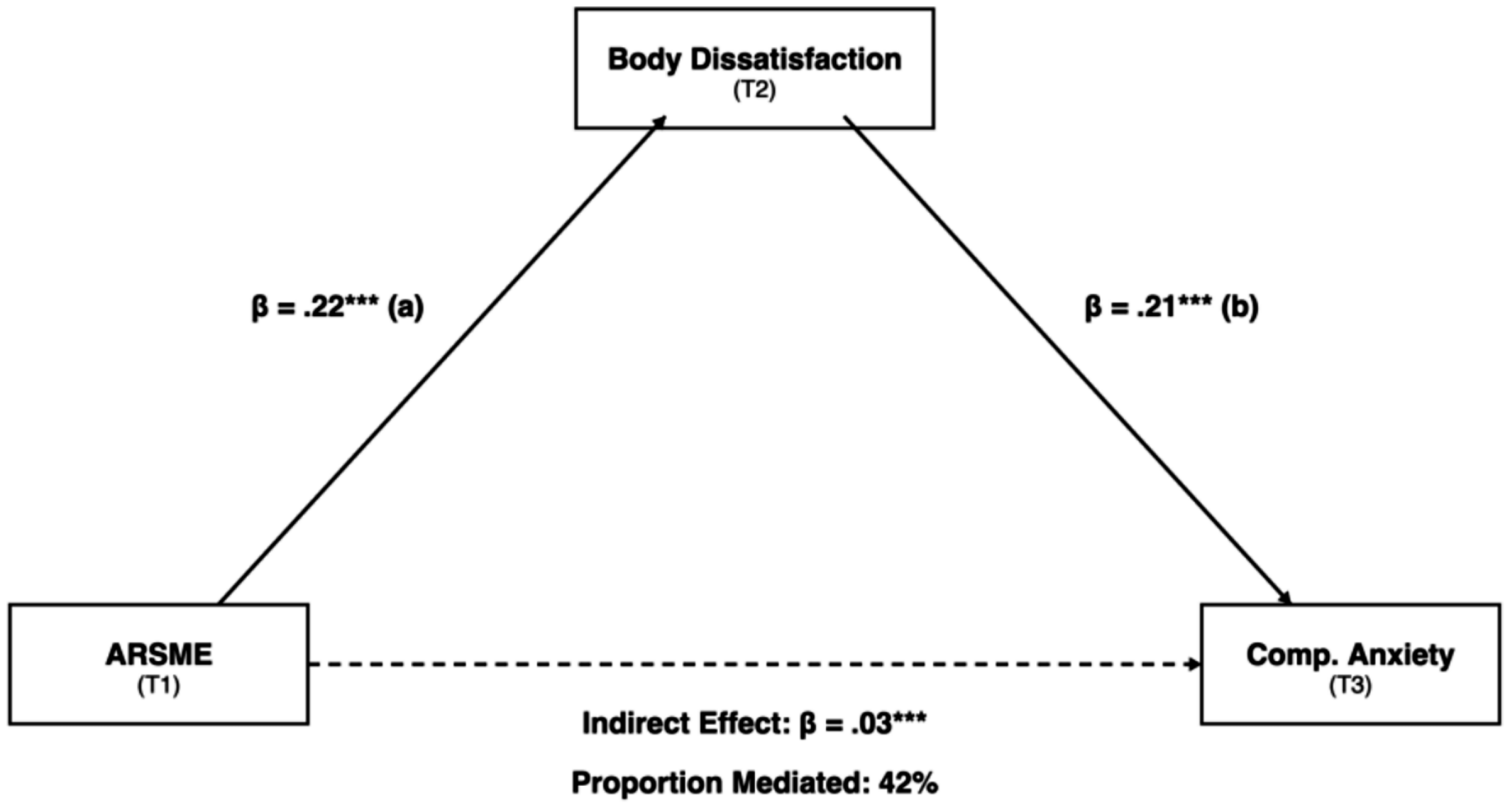
Mediation model: body dissatisfaction mediating ARSME–competitive anxiety. Indirect effect: *β* = 0.03, [0.01, 0.06]. Proportion mediated: 42%. ***p < 0.001.

Table 5 and Figure 5 present the overall mediation model. Body dissatisfaction significantly mediated the relationship between ARSME (T1) and competitive anxiety (T3). The indirect effect was significant [*β* = 0.03, (0.01, 0.06), *p* = 0.002], accounting for 42% of the total effect. The direct effect was non-significant (*β* = 0.04, *p* = 0.16), consistent with full mediation in the overall sample.

**Table 5:** Age-specific mediation effects.

Effect	Younger (14–16) β [95% CI]	Older (17–18) β [95% CI]
Path a: ARSME T1 → BD T2	0.22 [0.13, 0.31]***	0.13 [0.02, 0.24]*
Path b: BD T2 → CA T3	0.21 [0.12, 0.30]***	0.16 [0.05, 0.27]**
Indirect effect (a × b)	0.046 [0.02, 0.08]**	0.021 [0.00, 0.05]*
Direct effect (c’)	0.03 [−0.06, 0.12]	0.05 [−0.05, 0.15]
Proportion mediated	61%	30%

Bootstrap 10,000 resamples. Models control for sport type. *p < 0.05. **p < 0.01. ***p < 0.001.

**Figure 6:**
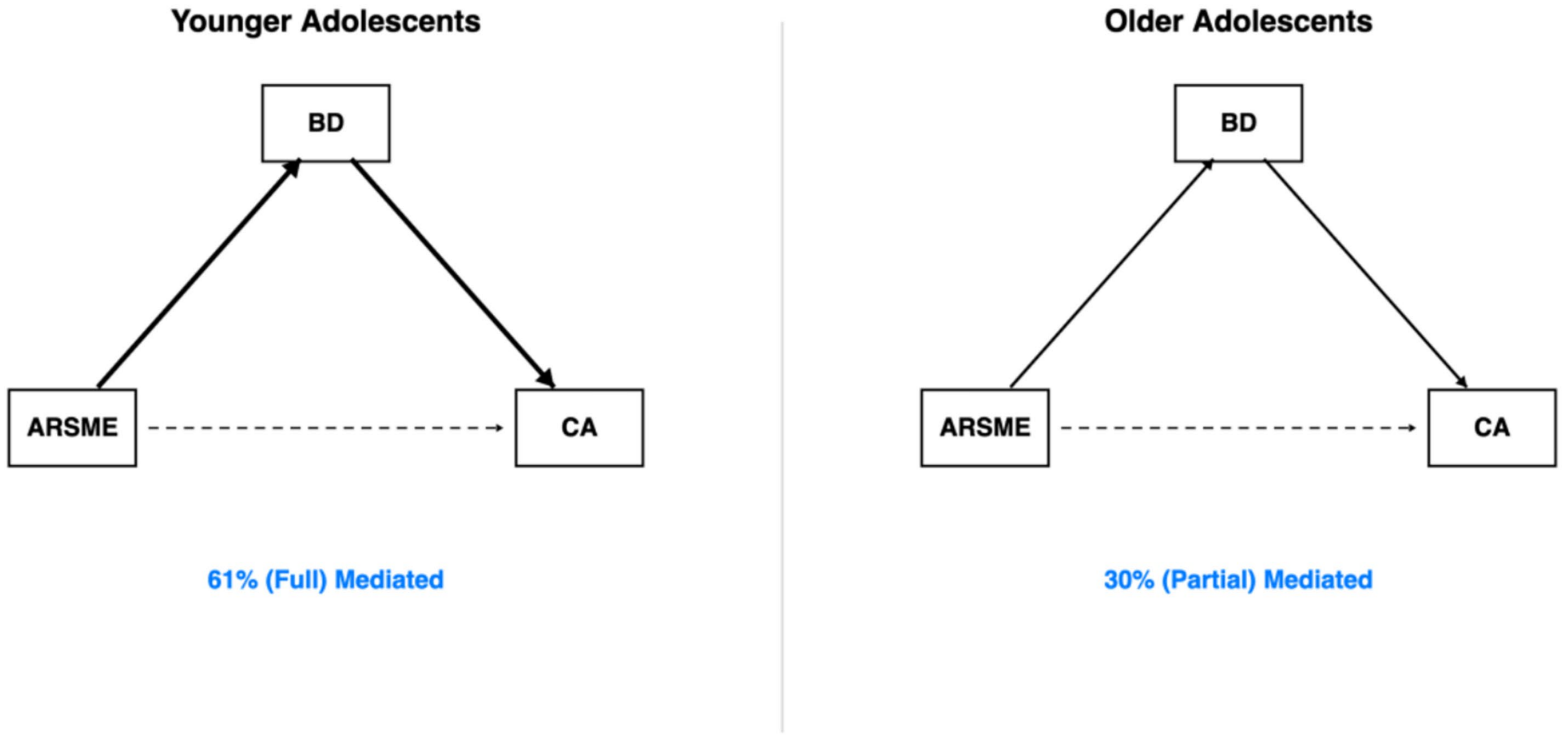
Age-specific mediation models. Left: younger adolescents (61% mediated, full mediation). Right: Older adolescents (30% mediated, partial mediation). Arrow thickness reflects effect magnitude.

Figure 6 compares age-specific mediation effects. In younger adolescents, the indirect effect of ARSME (T1) on competitive anxiety (T3) via body dissatisfaction (T2) was significant [*β* = 0.046, (0.02, 0.08), *p* = 0.002], explaining 61% of the total effect. The direct effect was non-significant (*β* = 0.03, *p* = 0.38), consistent with full mediation. In older adolescents, the indirect effect was smaller but still significant [*β* = 0.021, (0.00, 0.05), *p* = 0.042], explaining only 30% of the total effect. The significantly larger proportion of variance explained by the indirect path in younger adolescents (61% vs. 30%) supports Hypothesis 4.

### Random intercept cross-lagged panel model

The RI-CLPM with sport type controlled yielded acceptable fit: χ^2^(69) = 141.52, *p* < 0.001; CFI = 0.948, TLI = 0.932, RMSEA = 0.054. At the between-person level, stable trait-like associations emerged among ARSME, body dissatisfaction, and competitive anxiety (rs = 0.34–0.58, all ps < 0.001), indicating that individuals who generally engage more in appearance-focused social media also tend to experience higher body dissatisfaction and competitive anxiety. Importantly, these between-person correlations identify a “high-risk susceptible group” of athletes who exhibit chronically elevated levels across all three constructs, a profile that may require more intensive and sustained intervention than athletes with lower stable trait levels.

At the within-person level, results were consistent with the traditional CLPM. Deviations in ARSME at T1 predicted subsequent increases in body dissatisfaction at T2 (*β* = 0.11, *p* = 0.012), and body dissatisfaction deviations at T1 predicted increases in competitive anxiety at T2 (*β* = 0.10, *p* = 0.021). These within-person effects are particularly relevant for intervention design: they indicate that when an individual athlete experiences a period of elevated ARSME, specifically increased psychological preoccupation with online appearance feedback, they subsequently show elevated body dissatisfaction. From a clinical standpoint, the within-person effect sizes, though smaller than between-person estimates from the traditional CLPM, carry important practical significance. They reveal a malleable, dynamic process that is precisely the target of intervention: breaking the within-individual fluctuation chain from elevated ARSME to elevated BD to elevated CA. Athletes who exhibit chronically high stable trait levels across all three constructs (the “high-risk susceptible group” identified through between-person correlations) may require longer-term or more intensive intervention programs. The RI-CLPM findings thus delineate two complementary intervention goals: disrupting acute within-person fluctuation chains (suitable for universal or selective prevention) and addressing chronic high-trait risk profiles (requiring indicated, longer-term clinical attention). Specifically, for athletes exhibiting “state fluctuations” at the within-person level (i.e., short-term deviations from their personal average), universal, skill-oriented prevention programs such as mindfulness-based attention training and cognitive reappraisal skill development may be most appropriate, as these target the malleable cognitive processes that convert temporary spikes in ARSME into body dissatisfaction. In contrast, for athletes identified as “high trait-risk” at the between-person level, namely those with stable co-elevation across ARSME, body dissatisfaction, and competitive anxiety, longer-term, individualized clinical interventions centered on reshaping core self-perception and body schema may be required, as trait-level patterns are inherently less responsive to brief, skill-focused programs. This precision-intervention framework leverages the unique capacity of RI-CLPM to separate state-like and trait-like processes.

### Supplementary analyses

#### Continuous age moderation

To verify that findings were not artifacts of the categorical age grouping, we conducted supplementary analyses using latent moderated structural equations (LMS) with age as a continuous moderator. Results confirmed the primary findings: the Age × ARSME interaction significantly predicted body dissatisfaction at T2 (*β* = −0.09, *p* = 0.018), indicating that the ARSME → BD effect weakened with increasing age. No significant Age × BD interaction emerged for competitive anxiety (*β* = −0.02, *p* = 0.58), confirming that the BD → CA pathway operates similarly across the age continuum.

#### Sport type sensitivity analyses

**Figure 7:**
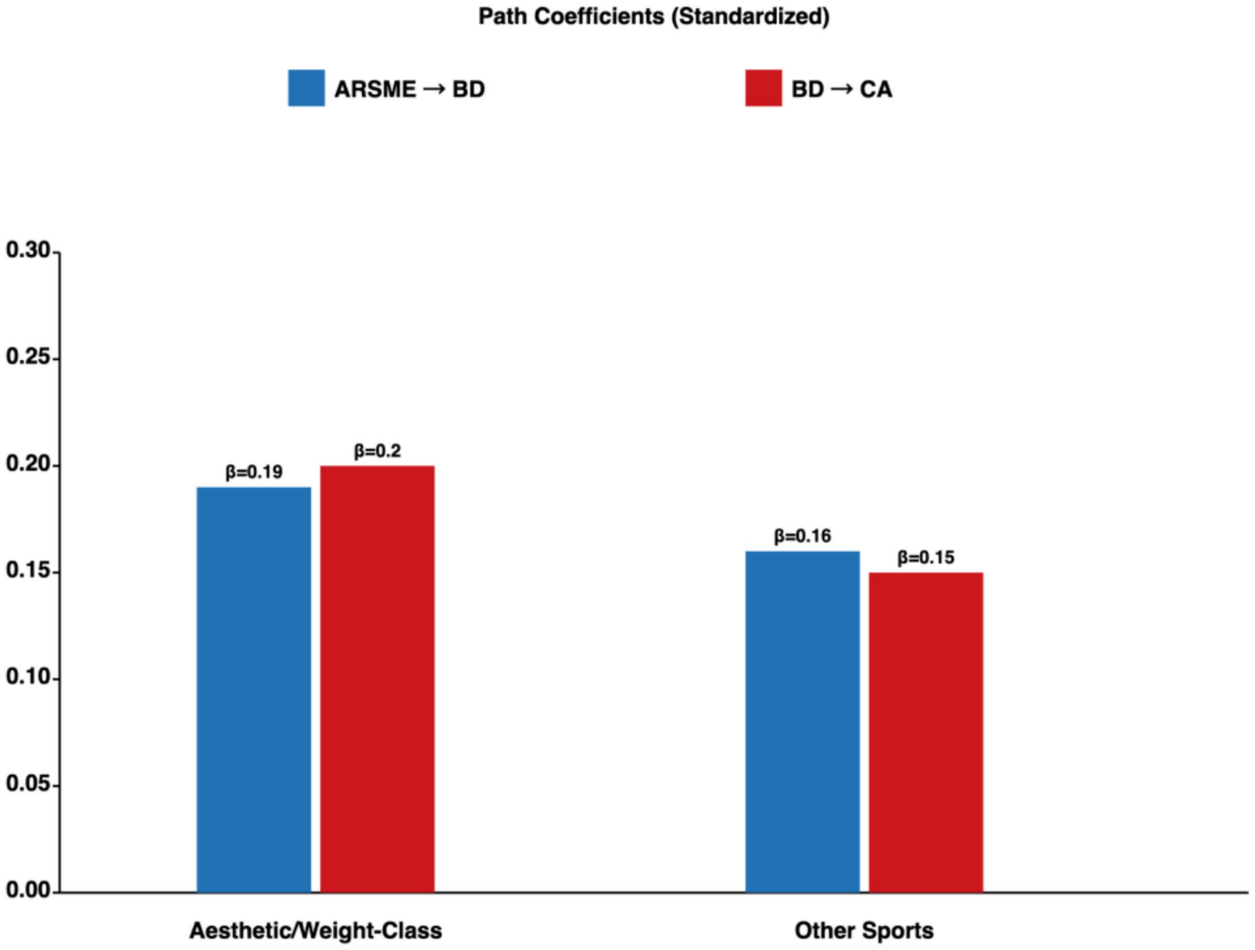
Sport type sensitivity analysis: path coefficients by athletic discipline. Pattern of cross-lagged effects consistent across aesthetic/weight-class and other sports. Three-way interaction (age × sport type × ARSME) non-significant (*p* = 0.34). *p < 0.05; **p < 0.01.

Table 6 summarizes sensitivity analyses. First, we estimated separate CLPM models for aesthetic/weight-class athletes (*n* = 120) and other athletes (*n* = 236). The pattern of cross-lagged effects was consistent across sport types (ARSME → BD: *β* = 0.15–0.19; BD → CA: *β* = 0.14–0.20; Figure 7). Second, we tested a three-way interaction (Age × Sport Type × ARSME) in predicting body dissatisfaction, which was non-significant (*p* = 0.34), indicating that the age moderation pattern did not differ by sport type.

**Table 6:** Summary of sensitivity analyses.

Analysis	Result	Conclusion
Sport type distribution by age	χ^2^ = 0.43, *p* = 0.510	No confounding
CLPM: Aesthetic/weight-class (*n* = 120)	ARSME→BD: *β* = 0.19; BD → CA: *β* = 0.20	Consistent pattern
CLPM: Other sports (*n* = 236)	ARSME→BD: *β* = 0.16; BD → CA: *β* = 0.15	Consistent pattern
3-way interaction (Age×Sport×ARSME)	*p* = 0.34	No differential moderation
Continuous age moderation (LMS)	Age×ARSME: *β* = −0.09, *p* = 0.018	Confirms categorical findings
Continuous: Age × BD → CA	*β* = −0.02, *p* = 0.58	BD → CA age-invariant

All sensitivity analyses support the robustness of primary findings. See Figure 7 for visualization of sport-type specific effects.

#### Component-level sensitivity analysis

Supplementary Table S1 presents results from a decomposed CLPM in which behavioral engagement (z-standardized daily social media time) and psychological investment (z-standardized ASMC scores) replaced the ARSME composite as separate predictors of body dissatisfaction. Critically, when both components were entered simultaneously in the full sample, the unique prospective effect of psychological investment on body dissatisfaction reached marginal significance from T2 to T3 (*β* = 0.08, *p* = 0.066), whereas behavioral engagement showed no significant unique effect at either interval (T1 → T2: *β* = −0.05, *p* = 0.30; T2 → T3: *β* = −0.06, *p* = 0.15). This pattern, whereby the cognitive-processing component approaches significance while the behavioral-exposure component does not, directly supports the theoretical perspective that it is the psychological processing of appearance-related content, rather than mere screen time, that drives body image disturbance (Valkenburg and Peter, 2013; Burnell et al., 2020). In age-stratified analyses, neither component reached statistical significance as an independent predictor in either age group, likely reflecting reduced statistical power when decomposing a composite measure into correlated sub-components within smaller subsamples. However, the direction of effects was informative: in the older group, psychological investment showed a positive trend (*β* = 0.09, *p* = 0.29 at T2 → T3) while behavioral time was near zero (*β* = −0.01, *p* = 0.88), consistent with the hypothesis that cognitive processing is more consequential than behavioral exposure.

## Discussion

This 12-month longitudinal study provides the first developmental examination of how appearance-related social media engagement relates to body dissatisfaction and competitive anxiety across adolescence in Chinese athletes. By applying a developmental psychopathology framework, controlling for sport type, and comparing younger (14–16 years) versus older (17–18 years) adolescents, we identified critical age-related differences in vulnerability to digital appearance pressures. Four key findings advance our theoretical and practical understanding.

### Developmental differences: younger adolescents as a high-risk group

Consistent with developmental theory, younger adolescents exhibited higher levels of ARSME, body dissatisfaction, and competitive anxiety across all assessment points (see Table 2 and Figure 1). These age differences (*d* = 0.24–0.45), while modest in magnitude, carry theoretical significance by identifying early-to-mid adolescence as a period of elevated vulnerability. These differences remained significant after controlling for sport type, confirming that they reflect genuine developmental effects rather than confounding by athletic discipline. This finding aligns with Somerville (2013) neurodevelopmental model positioning early adolescence as a “window of sensitivity” characterized by heightened responsiveness to social-evaluative stimuli, as well as research on the social reorientation that occurs during this period (Nelson et al., 2005; Blakemore and Mills, 2014).

The widening age gap in body dissatisfaction over the study period (d increasing from 0.29 at T1 to 0.45 at T3; Figure 2) is particularly noteworthy. This divergent trajectory, in which younger adolescents show increasing dissatisfaction while older adolescents stabilize, contrasts with cross-sectional studies that have reported linear age-related declines in body dissatisfaction (Bucchianeri et al., 2013) and highlights the importance of longitudinal designs for capturing developmental dynamics. The pattern may reflect both the accumulation of risk exposure in younger adolescents and the development of protective factors in older adolescents (Neumark-Sztainer et al., 2006).

Sport type exerted a significant main effect on body dissatisfaction (*β* = 0.12, *p* = 0.018), independent of age group. Athletes in aesthetic and weight-classified sports (gymnastics, diving, figure skating, wrestling, judo) exhibited higher body dissatisfaction regardless of developmental stage, consistent with established frameworks identifying these disciplines as contexts of heightened appearance pressure (Kong and Harris, 2015; Sundgot-Borgen and Torstveit, 2004). Although the Age × Sport Type interaction was non-significant (*p* = 0.34), the persistent main effect warrants clinical attention: practitioners should provide athletes in appearance-sensitive sports with proactive body image support irrespective of age.

### Age-moderated pathways: differential vulnerability at the “source”

A central contribution of this study is the demonstration that age moderates the prospective relationship between ARSME and body dissatisfaction (Table 4; Figure 4). At T1–T2, the effect was nearly twice as strong in younger adolescents (*β* = 0.22) compared to older adolescents (*β* = 0.13). This pattern was confirmed in supplementary analyses treating age as a continuous variable, ruling out artifacts of the categorical grouping. This finding extends Holland and Tiggemann (2016) meta-analytic conclusions about social media and body image by revealing developmentally specific effect sizes.

What accounts for this differential vulnerability? The dual-system model (Steinberg, 2010) offers a precise theoretical account: the period of 14–16 years is characterized by a social–emotional system that is highly active and sensitive to peer evaluation, while the cognitive control system (prefrontal cortex) has yet to fully mature. This developmental imbalance renders younger adolescents particularly reactive to appearance-related social feedback and relatively unable to regulate resulting emotional responses. We propose that the psychological investment component of ARSME, defined as the cognitive preoccupation with how one’s appearance is perceived online, operates differently across developmental stages. Younger adolescents, characterized by the ongoing maturation of the prefrontal cortex and heightened sensitivity to social evaluation (Casey et al., 2008; Somerville, 2013), may engage in more ruminative processing of appearance-related online feedback (Nolen-Hoeksema et al., 2008). This ruminative processing, in turn, may promote internalization of idealized appearance standards and subsequent body dissatisfaction (Fardouly et al., 2018). Older adolescents, having crossed critical neurodevelopmental thresholds (Luna et al., 2010), may be better equipped to cognitively reframe or dismiss such feedback, buffering its impact on body image (Silvers et al., 2015).

### Universal mechanisms: why the BD → CA pathway is age-invariant

Critically, age did not moderate the body dissatisfaction → competitive anxiety pathway. This finding has important theoretical implications. We interpret this as evidence that while the “sources” of body dissatisfaction are developmentally sensitive (i.e., younger adolescents are more vulnerable to developing body dissatisfaction from ARSME), the “downstream consequences” of established body dissatisfaction operate through relatively universal mechanisms. This apparent paradox of universal BD → CA mechanisms yet substantially different mediation proportions (61% vs. 30%) is resolved by the developmental cascade model (Masten and Cicchetti, 2010): the core principle of this model is that risk factors progressively “differentiate” and “diffuse” across multiple pathways as individuals develop. For younger adolescents, ARSME operates through a relatively streamlined, dominant risk cascade: ARSME → BD → CA. For older adolescents, ARSME simultaneously activates this same BD → CA cascade alongside additional parallel risk pathways, including performance-based social comparison and impression management demands, thereby diluting the relative mediating contribution of BD without diminishing its universal role as a risk mechanism. Crucially, it is the developmental multiplication of risk pathways, rather than any weakening of the BD → CA link itself, that accounts for the differential mediation proportions.

Why might this be the case? Body dissatisfaction, once established, represents a fundamental disruption to the athlete’s relationship with their physical self as the very instrument of their competitive performance. This disruption activates anxiety through common psychological pathways that are unlikely to differ by developmental stage: (1) heightened self-consciousness during competition, as athletes become preoccupied with how their bodies appear to others rather than focusing on performance cues (Leary, 1992; Sabiston et al., 2007); (2) attentional interference, as appearance-monitoring competes with task-relevant focus consistent with processing efficiency theory (Eysenck et al., 2007); and (3) undermined confidence in physical capabilities, as the body is perceived as inadequate rather than as a competent tool for athletic achievement (Hausenblas and Fallon, 2006).

For intervention purposes, this finding suggests a critical insight: while *prevention* efforts should prioritize younger adolescents (given their heightened vulnerability to developing body dissatisfaction from ARSME), *treatment* approaches targeting existing body image concerns may be equally effective across the adolescent age range.

### Partial mediation in older adolescents: alternative mechanisms

The finding that body dissatisfaction mediated a larger proportion of the ARSME–CA relationship in younger (61%) versus older (30%) adolescents (Table 5; Figure 6) raises an important question: through what alternative mechanisms does ARSME affect competitive anxiety in older adolescents?

We propose several possibilities grounded in developmental theory. First, older adolescents may experience social comparison processes that extend beyond body image to encompass performance-related comparisons (Vogel et al., 2014). Social media platforms increasingly showcase athletes’ training achievements, competition results, and career milestones. Given that older adolescents are closer to critical career transitions such as collegiate recruitment or professional selection, exposure to peers’ accomplishments may trigger performance-based social comparison anxiety that bypasses body image concerns. This interpretation aligns with research showing that upward social comparison processes intensify during late adolescence as future-oriented thinking matures (Steinberg et al., 2009).

Second, older athletes may experience heightened pressures related to maintaining a public athletic persona on social media. Unlike younger adolescents whose online presence may be more appearance-focused, older athletes may face expectations to project competence, confidence, and success, thereby creating anxiety about the gap between their curated online image and their actual competitive uncertainties (Geurin-Eagleman and Burch, 2016). This “impression management” burden represents a distinct pathway from body dissatisfaction.

Third, the diluted mediating role of body dissatisfaction in older adolescents may reflect the emergence of multiple parallel pathways rather than reduced overall vulnerability. This interpretation aligns with cascading models of developmental psychopathology (Masten and Cicchetti, 2010), which propose that risk processes become more differentiated and complex across development. For younger adolescents, the ARSME → BD → CA cascade may represent a dominant, streamlined risk pathway. For older adolescents, this pathway persists but operates alongside additional mechanisms, reducing its proportional contribution.

### Within-person processes: implications for intervention targets

The RI-CLPM results provide compelling evidence that the observed associations reflect genuine within-person processes rather than stable between-person differences. When an individual athlete experiences a period of increased appearance-focused social media engagement, specifically elevated psychological preoccupation with online appearance feedback, they subsequently show elevated body dissatisfaction. This within-person evidence strengthens causal inference and has critical implications for intervention design (Keijsers, 2016).

Specifically, the results suggest that targeting the psychological investment component of ARSME (i.e., cognitive preoccupation with online appearance feedback) may be more impactful than simply reducing screen time. An athlete who spends the same amount of time on social media but engages with appearance-related content less ruminatively may experience reduced body image impacts (Kayser et al., 2019). This aligns with emerging evidence that it is not mere exposure to social media but rather the cognitive processing of that content that drives psychological outcomes (Kayser et al., 2019).

The supplementary sensitivity analysis decomposing ARSME into its behavioral and psychological components provides additional, albeit preliminary, insight. In the full sample, the unique effect of psychological investment (ASMC) on body dissatisfaction reached marginal significance (*β* = 0.08, *p* = 0.066), whereas behavioral engagement (daily time) did not (*β* = −0.06, *p* = 0.15). Although neither component reached conventional significance when entered simultaneously (an expected consequence of decomposing correlated composite sub-components), the consistent directional pattern supports the theoretical argument that cognitive preoccupation with online appearance feedback, rather than mere behavioral exposure, is the primary driver of body image disturbance. This finding aligns with Valkenburg and Peter’s (2013) differential susceptibility model. Importantly, this empirical pattern elevates the intervention recommendation of cognitive reappraisal training from theoretical speculation to evidence-supported practice: the data suggest that an athlete who spends the same amount of time on social media but engages with appearance-related content less ruminatively may experience reduced body image impacts, providing a direct empirical basis for prioritizing cognitive-reappraisal-focused interventions over mere screen time reduction.

### Practical implications: toward developmentally informed, cognitively targeted interventions

Our findings point to several evidence-based intervention recommendations that differ in focus depending on the target population.

#### For younger adolescents (14–16 years): prevention-focused approaches

Given their heightened vulnerability to developing body dissatisfaction from ARSME, younger athletes should be prioritized for preventive interventions. However, our findings suggest that such interventions should move beyond simple screen time reduction to target the psychological investment component of ARSME, with a focus on the tendency to ruminate on and internalize appearance-related online feedback.

Evidence-based strategies include: (1) Cognitive reappraisal training, teaching athletes to recognize and challenge automatic negative thoughts triggered by appearance-related social media content (e.g., “That photo is edited and doesn’t represent reality”; “My worth as an athlete is not determined by likes or comments”) (McLean et al., 2017); (2) Metacognitive awareness, helping athletes recognize when they are engaging in appearance-focused rumination and develop “detachment” skills to disengage from such thinking patterns (Wells, 2011); and (3) Functionality-focused body appreciation, shifting attention from how the body looks to what the body can do (Alleva et al., 2015), which may be particularly effective for athletes whose bodies serve instrumental purposes.

#### For older adolescents (17–18 years): addressing multiple pathways

While body image interventions remain relevant for older athletes, the partial mediation findings suggest that additional intervention targets may be warranted. These include: (1) Performance-based social comparison management, helping athletes develop realistic frameworks for evaluating their own progress without excessive comparison to curated online portrayals of peers’ achievements; and (2) Authentic self-presentation skills, reducing the burden of maintaining idealized online personas by promoting more genuine online engagement (Reinecke and Trepte, 2014).

#### For coaches and sport psychologists

Practitioners should be attuned to age-related differences in vulnerability. Younger athletes (14–16) showing elevations in appearance-focused social media use may benefit from proactive cognitive-focused interventions. Team cultures that de-emphasize appearance and celebrate diverse body types and performance styles may provide additional protection, particularly for younger athletes (Voelker et al., 2014).

### Cultural considerations

Our findings should be interpreted within the Chinese cultural context. Traditional Chinese beauty ideals and the cultural emphasis on “face” may amplify appearance-related pressures in distinctive ways (Jung and Lee, 2006; Qi, 2011). Notably, the concept of “face,” reflecting the deep cultural concern with how one is perceived and evaluated by others, may have a specific amplifying effect on the psychological investment component of ARSME identified in our sensitivity analysis. Chinese adolescents’ culturally heightened sensitivity to social evaluation may intensify cognitive preoccupation with how their appearance is perceived online, promoting ruminative processing of appearance-related feedback beyond what would be expected from universal developmental processes alone. This cultural amplification of psychological investment, rather than of behavioral screen time per se, represents a unique contribution of the Chinese context that extends beyond universal developmental patterns and underscores the particular relevance of cognitive reappraisal interventions for this population. However, the fundamental pattern of developmental differences in vulnerability to appearance-focused media likely reflects universal neurodevelopmental processes, even as specific manifestations may vary across cultures (Chen and French, 2008). Cross-cultural replication is needed to establish the generalizability of these findings.

### Limitations and future directions

Several limitations warrant consideration. First, while our longitudinal design supports temporal ordering, experimental manipulation of ARSME would strengthen causal claims. Second, all measures were self-reported; future research should incorporate objective digital tracking data and multi-informant assessments. Third, our categorical age grouping, while theoretically grounded in neurodevelopmental literature, involves some arbitrariness; the supplementary continuous age analyses partially address this concern. Fourth, we did not directly assess the alternative mechanisms proposed for older adolescents (e.g., performance-based social comparison, impression management anxiety); these untested pathways should be treated as theoretically-motivated hypotheses requiring direct empirical evaluation in future research. Fifth, the sample is drawn exclusively from four sports schools in Jiangxi Province, which limits generalizability even within China. Findings should be interpreted with caution when extrapolating to athletes in other Chinese provinces or different types of sports institutions. Additionally, cross-cultural replication with Western samples is needed. Sixth, the supplementary sensitivity analysis decomposing ARSME into behavioral and psychological components was limited by reduced statistical power inherent in entering correlated sub-components simultaneously; future research with larger samples should replicate this decomposition.

Future research should: (1) directly test whether interventions targeting cognitive reappraisal of online feedback are more effective than screen time reduction; (2) conduct adequately powered sensitivity analyses entering behavioral engagement and psychological investment as independent predictors to determine which component drives prospective effects; (3) examine the specific mechanisms through which ARSME affects competitive anxiety in older adolescents; (4) identify protective factors that buffer younger adolescents against digital appearance pressures, including media literacy, parental communication, and athletic identity; and (5) investigate whether findings generalize to non-athlete adolescent populations.

### Conclusion

This study provides novel developmental evidence that appearance-related social media engagement poses particular risks for younger adolescent athletes (14–16 years), who show heightened vulnerability to developing body dissatisfaction from digital appearance pressures. Critically, while the “source” of body dissatisfaction (ARSME) shows age-moderated effects, the “consequence” of established body dissatisfaction (competitive anxiety) operates through universal mechanisms across developmental stages. These findings position early-to-mid adolescence as a sensitive period warranting targeted prevention efforts. Developmentally informed interventions that target the cognitive processing of appearance-related online feedback, rather than mere screen time, are needed to protect young athletes’ psychological health and competitive potential.

## Data Availability

The raw data supporting the conclusions of this article will be made available by the authors, without undue reservation.

## References

[ref1] Albert D. Steinberg L. (2011). Peer influences on adolescent risk behavior. Inhibitory Control Drug Abuse Prevention. *From Research to Translation*, (Eds.) M. T. Bardo, D. H. Fishbein, and R. Milich (New York, NY: Springer), 211–228. doi: 10.1007/978-1-4419-1268-8_11

[ref2] Alleva J. M. Martijn C. Van Breukelen G. J. Jansen A. Karos K. (2015). Expand your horizon: a programme that improves body image and reduces self-objectification by training women to focus on body functionality. Body Image 15, 81–89. doi: 10.1016/j.bodyim.2015.07.001, 26280376

[ref3] Anderson M. Jiang J. (2018). Teens, social media & technology 2018. Pew Research Center 31, 1673–1689.

[ref4] Blakemore S. J. Choudhury S. (2006). Development of the adolescent brain: implications for executive function and social cognition. J. Child Psychol. Psychiatry 47, 296–312. doi: 10.1111/j.1469-7610.2006.01611.x, 16492261

[ref5] Blakemore S.-J. Mills K. L. (2014). Is adolescence a sensitive period for sociocultural processing? Annu. Rev. Psychol. 65, 187–207. doi: 10.1146/annurev-psych-010213-115202, 24016274

[ref9001] Bucchianeri M. M. Arikian A. J. Hannan P. J. Eisenberg M. E. Neumark-Sztainer D. (2013). Body dissatisfaction from adolescence to young adulthood: findings from a 10-year longitudinal study. Body Image 10, 1–7. doi: 10.1016/j.bodyim.2012.09.001 23084464 PMC3814026

[ref6] Burnell K. George M. J. Underwood M. K. (2020). Browsing different Instagram profiles and associations with psychological well-being. Front. Hum. Dyn. 2:585518. doi: 10.3389/fhumd.2020.585518

[ref7] Casey B. J. Getz S. Galvan A. (2008). The adolescent brain. Dev. Rev. 28, 62–77. doi: 10.1016/j.dr.2007.08.003, 18688292 PMC2500212

[ref8] Chen X. French D. C. (2008). Children's social competence in cultural context. Annu. Rev. Psychol. 59, 591–616. doi: 10.1146/annurev.psych.59.103006.093606, 18154504

[ref9] Chen H. Jackson T. Huang X. (2006). The negative physical self scale: initial development and validation in samples of Chinese adolescents and young adults. Body Image 3, 401–412. doi: 10.1016/j.bodyim.2006.07.005, 18089244

[ref10] Choukas-Bradley S. Nesi J. Widman L. Galla B. M. (2020). The appearance-related social media consciousness scale: development and validation with adolescents. Body Image 33, 164–174. doi: 10.1016/j.bodyim.2020.02.017, 32193170

[ref11] Cicchetti D. Rogosch F. A. (2002). A developmental psychopathology perspective on adolescence. J. Consult. Clin. Psychol. 70:6. doi: 10.1037/0022-006x.70.1.6 11860057

[ref12] Cnnic (2018). The 51st Statistical Report on Internet Development in China. Beijing, China: China Internet Network Information Center (Cnnic).

[ref13] Collins L. M. (2006). Analysis of longitudinal data: the integration of theoretical model, temporal design, and statistical model. Annu. Rev. Psychol. 57, 505–528. doi: 10.1146/annurev.psych.57.102904.190146, 16318605

[ref14] Crone E. A. Dahl R. E. (2012). Understanding adolescence as a period of social–affective engagement and goal flexibility. Nat. Rev. Neurosci. 13, 636–650. doi: 10.1038/nrn3313, 22903221

[ref15] Dahl R. E. (2004). Adolescent brain development: a period of vulnerabilities and opportunities. Keynote address. Ann. N. Y. Acad. Sci. 1021, 1–22. doi: 10.1196/annals.1308.001, 15251869

[ref16] Dahl R. E. Allen N. B. Wilbrecht L. Suleiman A. B. (2018). Importance of investing in adolescence from a developmental science perspective. Nature 554, 441–450. doi: 10.1038/nature25770, 29469094

[ref17] De Bruin A. K. Oudejans R. R. Bakker F. C. (2007). Dieting and body image in aesthetic sports: a comparison of Dutch female gymnasts and non-aesthetic sport participants. Psychol. Sport Exerc. 8, 507–520. doi: 10.1016/j.psychsport.2006.10.002

[ref18] Deci E. L. Ryan R. M. (2000). The “what” and “why” of goal pursuits: human needs and the self-determination of behavior. Psychol. Inq. 11, 227–268. doi: 10.1207/s15327965pli1104

[ref19] Enders C. K. Bandalos D. L. (2001). The relative performance of full information maximum likelihood estimation for missing data in structural equation models. Struct. Equ. Model. 8, 430–457. doi: 10.1207/S15328007SEM0803_5

[ref9002] Erikson E. H. (1968). Identity: Youth and Crisis. New York: W. W. Norton & Company.

[ref20] Eysenck M. W. Calvo M. G. (1992). Anxiety and performance: the processing efficiency theory. Cogn. Emot. 6, 409–434. doi: 10.1080/02699939208409696

[ref21] Eysenck M. W. Derakshan N. Santos R. Calvo M. G. (2007). Anxiety and cognitive performance: attentional control theory. Emotion 7:336. doi: 10.1037/1528-3542.7.2.336, 17516812

[ref22] Fardouly J. Vartanian L. R. (2016). Social media and body image concerns: current research and future directions. Curr. Opin. Psychol. 9, 1–5. doi: 10.1016/j.copsyc.2015.09.005

[ref23] Fardouly J. Willburger B. K. Vartanian L. R. (2018). Instagram use and young women’s body image concerns and self-objectification: testing mediational pathways. New Media Soc. 20, 1380–1395. doi: 10.1177/1461444817694499

[ref24] Faul F. Erdfelder E. Buchner A. Lang A.-G. (2009). Statistical power analyses using G* power 3.1: tests for correlation and regression analyses. Behav. Res. Methods 41, 1149–1160. doi: 10.3758/brm.41.4.1149, 19897823

[ref25] Francisco R. Narciso I. Alarcao M. (2013). Individual and relational risk factors for the development of eating disorders in adolescent aesthetic athletes and general adolescents. Eat. Weight Disord. 18, 403–411. doi: 10.1007/s40519-013-0055-6, 23943379

[ref26] Fredrickson B. L. Roberts T. A. (1997). Objectification theory: toward understanding women's lived experiences and mental health risks. Psychol. Women Q. 21, 173–206. doi: 10.1111/j.1471-6402.1997.tb00108.x

[ref27] Gattario K. H. Frisén A. (2019). From negative to positive body image: men’s and women’s journeys from early adolescence to emerging adulthood. Body Image 28, 53–65. doi: 10.1016/j.bodyim.2018.12.002, 30583277

[ref28] Geurin-Eagleman A. N. Burch L. M. (2016). Communicating via photographs: a gendered analysis of Olympic athletes’ visual self-presentation on Instagram. Sport Manag. Rev. 19, 133–145. doi: 10.1016/j.smr.2015.03.002

[ref29] Giedd J. N. Blumenthal J. Jeffries N. O. Castellanos F. X. Liu H. Zijdenbos A. . (1999). Brain development during childhood and adolescence: a longitudinal MRI study. Nat. Neurosci. 2, 861–863. doi: 10.1038/13158, 10491603

[ref30] Grabe S. Ward L. M. Hyde J. S. (2008). The role of the media in body image concerns among women: a meta-analysis of experimental and correlational studies. Psychol. Bull. 134:460. doi: 10.1037/0033-2909.134.3.460, 18444705

[ref31] Graber J. A. Nichols T. R. Brooks-Gunn J. (2010). Putting pubertal timing in developmental context: implications for prevention. Dev. Psychobiol. 52, 254–262. doi: 10.1002/dev.20438, 20196112

[ref32] Grossbard J. R. Smith R. E. Smoll F. L. Cumming S. P. (2009). Competitive anxiety in young athletes: differentiating somatic anxiety, worry, and concentration disruption. Anxiety Stress Coping 22, 153–166. doi: 10.1080/10615800802020643, 18937102

[ref33] Hamaker E. L. Kuiper R. M. Grasman R. P. (2015). A critique of the cross-lagged panel model. Psychol. Methods 20, 102–116. doi: 10.1037/a0038889, 25822208

[ref34] Harter S. (2015). The Construction of the Self: Developmental and Sociocultural Foundations. New York, NY: Guilford Publications.

[ref35] Hausenblas H. A. Fallon E. A. (2006). Exercise and body image: a meta-analysis. Psychol. Health 21, 33–47. doi: 10.1080/14768320500105270

[ref36] Holland G. Tiggemann M. (2016). A systematic review of the impact of the use of social networking sites on body image and disordered eating outcomes. Body Image 17, 100–110. doi: 10.1016/j.bodyim.2016.02.008, 26995158

[ref37] Hu L. T. Bentler P. M. (1999). Cutoff criteria for fit indexes in covariance structure analysis: conventional criteria versus new alternatives. Struct. Equ. Model. 6, 1–55.

[ref38] Jung J. Lee S. H. (2006). Cross-cultural comparisons of appearance self-schema, body image, self-esteem, and dieting behavior between Korean and US women. Fam. Consum. Sci. Res. J. 34, 350–365. doi: 10.1177/1077727X06286419

[ref39] Kayser J. Tenke C. E. Svob C. Gameroff M. J. Miller L. Skipper J. . (2019). Family risk for depression and prioritization of religion or spirituality: early neurophysiological modulations of motivated attention. Front. Hum. Neurosci. 13:436. doi: 10.3389/fnhum.2019.00436, 31920595 PMC6927907

[ref40] Keery H. Van Den Berg P. Thompson J. K. (2004). An evaluation of the tripartite influence model of body dissatisfaction and eating disturbance with adolescent girls. Body Image 1, 237–251. doi: 10.1016/j.bodyim.2004.03.001, 18089156

[ref41] Keijsers L. (2016). Parental monitoring and adolescent problem behaviors: how much do we really know? Int. J. Behav. Dev. 40, 271–281. doi: 10.1177/0165025415592515

[ref42] Klein A. Moosbrugger H. (2000). Maximum likelihood estimation of latent interaction effects with the LMS method. Psychometrika 65, 457–474. doi: 10.1007/bf02296338

[ref43] Klimstra T. A. Hale Iii W. W. Raaijmakers Q. A. Branje S. J. Meeus W. H. (2010). Identity formation in adolescence: change or stability? J. Youth Adolesc. 39, 150–162. doi: 10.1007/s10964-009-9401-4, 20084561 PMC2807933

[ref44] Kline R. B. (2023). Principles and Practice of Structural Equation Modeling. New York, NY: Guilford publications.

[ref45] Kong P. Harris L. M. (2015). The sporting body: body image and eating disorder symptomatology among female athletes from leanness focused and nonleanness focused sports. J. Psychol. 149, 141–160. doi: 10.1080/00223980.2013.846291, 25511202

[ref46] Kroger J. Martinussen M. Marcia J. E. (2010). Identity status change during adolescence and young adulthood: a meta-analysis. J. Adolesc. 33, 683–698. doi: 10.1016/j.adolescence.2009.11.002, 20004962

[ref47] Leary M. R. (1992). Self-presentational processes in exercise and sport. J. Sport Exerc. Psychol. 14, 339–351. doi: 10.1123/jsep.14.4.339

[ref48] Little T. D. (2013). The Oxford Handbook of Quantitative Methods. New York, NY: Oxford University Press.

[ref49] Livingstone S. (2014). Developing social media literacy: how children learn to interpret risky opportunities on social network sites. Commun. Eur. J. Commun. Res. 39, 283–303. doi: 10.1515/commun-2014-0113

[ref50] Luna B. Padmanabhan A. O’hearn K. (2010). What has fmri told us about the development of cognitive control through adolescence? Brain Cogn. 72, 101–113. doi: 10.1016/j.bandc.2009.08.005, 19765880 PMC2815087

[ref51] Luo W. (2013). Aching for the altered body: beauty economy and Chinese women's consumption of cosmetic surgery. Womens Stud. Int. Forum 38, 1–10. doi: 10.1016/j.wsif.2013.01.013

[ref52] Mackinnon D. P. Lockwood C. M. Hoffman J. M. West S. G. Sheets V. (2002). A comparison of methods to test mediation and other intervening variable effects. Psychol. Methods 7:83. doi: 10.1037/1082-989x.7.1.83, 11928892 PMC2819363

[ref53] Martens R. Vealey R. S. Burton D. (1990). Competitive Anxiety in Sport. Champaign, IL: Human Kinetics.

[ref54] Masten A. S. Cicchetti D. (2010). Developmental cascades. Dev. Psychopathol. 22, 491–495. doi: 10.1017/s0954579410000222, 20576173

[ref55] Mclean S. A. Paxton S. J. Wertheim E. H. (2016). The role of media literacy in body dissatisfaction and disordered eating: a systematic review. Body Image 19, 9–23. doi: 10.1016/j.bodyim.2016.08.002, 27572000

[ref56] Mclean S. A. Wertheim E. H. Masters J. Paxton S. J. (2017). A pilot evaluation of a social media literacy intervention to reduce risk factors for eating disorders. Int. J. Eat. Disord. 50, 847–851. doi: 10.1002/eat.22708, 28370321

[ref57] Meeus W. (2011). The study of adolescent identity formation 2000–2010: a review of longitudinal research. J. Res. Adolesc. 21, 75–94. doi: 10.1111/j.1532-7795.2010.00716.x

[ref58] Mendle J. Turkheimer E. Emery R. E. (2007). Detrimental psychological outcomes associated with early pubertal timing in adolescent girls. Dev. Rev. 27, 151–171. doi: 10.1016/j.dr.2006.11.001, 20740062 PMC2927128

[ref59] Mingoia J. Hutchinson A. D. Wilson C. Gleaves D. H. (2017). The relationship between social networking site use and the internalization of a thin ideal in females: a meta-analytic review. Front. Psychol. 8:1351. doi: 10.3389/fpsyg.2017.01351, 28824519 PMC5545754

[ref60] Nelson E. E. Leibenluft E. Mcclure E. B. Pine D. S. (2005). The social re-orientation of adolescence: a neuroscience perspective on the process and its relation to psychopathology. Psychol. Med. 35, 163–174. doi: 10.1017/S0033291704003915, 15841674

[ref61] Nesi J. Prinstein M. J. (2015). Using social media for social comparison and feedback-seeking: gender and popularity moderate associations with depressive symptoms. J. Abnorm. Child Psychol. 43, 1427–1438. doi: 10.1007/s10802-015-0020-0, 25899879 PMC5985443

[ref62] Neumark-Sztainer D. Paxton S. J. Hannan P. J. Haines J. Story M. (2006). Does body satisfaction matter? Five-year longitudinal associations between body satisfaction and health behaviors in adolescent females and males. J. Adolesc. Health 39, 244–251. doi: 10.1016/j.jadohealth.2005.12.001, 16857537

[ref63] Nolen-Hoeksema S. Wisco B. E. Lyubomirsky S. (2008). Rethinking rumination. Perspect. Psychol. Sci. 3, 400–424. doi: 10.1111/j.1745-6924.2008.00088.x, 26158958

[ref64] Perloff R. M. (2014). Social media effects on young women’s body image concerns: theoretical perspectives and an agenda for research. Sex Roles 71, 363–377. doi: 10.1007/s11199-014-0384-6

[ref65] Podsakoff P. M. Mackenzie S. B. Lee J.-Y. Podsakoff N. P. (2003). Common method biases in behavioral research: a critical review of the literature and recommended remedies. J. Appl. Psychol. 88, 879–903. doi: 10.1037/0021-9010.88.5.879, 14516251

[ref66] Putnick D. L. Bornstein M. H. (2016). Measurement invariance conventions and reporting: the state of the art and future directions for psychological research. Dev. Rev. 41, 71–90. doi: 10.1016/j.dr.2016.06.004, 27942093 PMC5145197

[ref67] Qi X. (2011). Face: a Chinese concept in a global sociology. J. Sociol. 47, 279–295. doi: 10.1177/1440783311407692

[ref68] Reinecke L. Trepte S. (2014). Authenticity and well-being on social network sites: a two-wave longitudinal study on the effects of online authenticity and the positivity bias in SNS communication. Comput. Human Behav. 30, 95–102. doi: 10.1016/j.chb.2013.07.030

[ref69] Rideout V. (2015). The Common Sense Census: Media Use by Tweens and Teens. San Francisco, CA: Common Sense Media.

[ref70] Rodgers R. F. Slater A. Gordon C. S. Mclean S. A. Jarman H. K. Paxton S. J. (2020). A biopsychosocial model of social media use and body image concerns, disordered eating, and muscle-building behaviors among adolescent girls and boys. J. Youth Adolesc. 49, 399–409. doi: 10.1007/s10964-019-01190-0, 31907699

[ref71] Rosseel Y. (2012). Lavaan: an R package for structural equation modeling. J. Stat. Softw. 48, 1–36. doi: 10.18637/jss.v048.i02

[ref72] Ryan R. M. Deci E. L. (2024). Self-Determination Theory. Encyclopedia of Quality of Life and Well-Being Research. Cham, Switzerland: Springer International Publishing.

[ref73] Sabiston C. Sedgwick W. Crocker P. Kowalski K. Mack D. (2007). Social physique anxiety in adolescence: an exploration of influences, coping strategies, and health behaviors. J. Adolesc. Res. 22, 78–101. doi: 10.1177/0743558406294628

[ref74] Saiphoo A. N. Vahedi Z. (2019). A meta-analytic review of the relationship between social media use and body image disturbance. Comput. Human Behav. 101, 259–275. doi: 10.1016/j.chb.2019.07.028

[ref75] Satorra A. Bentler P. M. (2001). A scaled difference chi-square test statistic for moment structure analysis. Psychometrika 66, 507–514. doi: 10.1007/bf02296192 PMC2905175 20640194

[ref76] Sebastian C. Viding E. Williams K. D. Blakemore S.-J. (2010). Social brain development and the affective consequences of ostracism in adolescence. Brain Cogn. 72, 134–145. doi: 10.1016/j.bandc.2009.06.008, 19628323

[ref77] Selig J. P. Little T. D. (2012). Autoregressive and Cross-Lagged panel Analysis for Longitudinal Data. New York, NY: The Guilford Press.

[ref78] Silvers J. A. Shu J. Hubbard A. D. Weber J. Ochsner K. N. (2015). Concurrent and lasting effects of emotion regulation on amygdala response in adolescence and young adulthood. Dev. Sci. 18, 771–784. doi: 10.1111/desc.12260, 25439326 PMC4459932

[ref79] Slater A. Tiggemann M. (2015). Media exposure, extracurricular activities, and appearance-related comments as predictors of female adolescents’ self-objectification. Psychol. Women Q. 39, 375–389. doi: 10.1177/0361684314554606

[ref80] Smith R. E. Smoll F. L. Cumming S. P. Grossbard J. R. (2006). Measurement of multidimensional sport performance anxiety in children and adults: the sport anxiety Scale-2. J. Sport Exerc. Psychol. 28, 479–501. doi: 10.1123/jsep.28.4.479

[ref81] Somerville L. H. (2013). The teenage brain: sensitivity to social evaluation. Curr. Dir. Psychol. Sci. 22, 121–127. doi: 10.1177/0963721413476512, 24761055 PMC3992953

[ref82] Sroufe L. A. Rutter M. (1984). The domain of developmental psychopathology. Child Dev. 55, 17–29. doi: 10.2307/1129832, 6705619

[ref83] Steinberg L. (2010). A dual systems model of adolescent risk-taking. Dev. Psychobiol. 52, 216–224. doi: 10.1002/dev.20445, 20213754

[ref930] Steinberg L. (2017). “A social neuroscience perspective on adolescent risk-taking,” in Biosocial Theories of Crime, eds. A. Walsh and K. M. Beaver (New York: Routledge), 435–455.

[ref84] Steinberg L. Graham S. O’brien L. Woolard J. Cauffman E. Banich M. (2009). Age differences in future orientation and delay discounting. Child Dev. 80, 28–44. doi: 10.1111/j.1467-8624.2008.01244.x 19236391

[ref85] Sundgot-Borgen J. Torstveit M. K. (2004). Prevalence of eating disorders in elite athletes is higher than in the general population. Clin. J. Sport Med. 14, 25–32. doi: 10.1097/00042752-200401000-00005, 14712163

[ref86] Susman E. J. Dorn L. D. (2012). “Puberty: its role in development,” in Handbook of Psychology, Second Edition, 6, (Hoboken, NJ: John Wiley & Sons).

[ref87] Thompson J. K. Heinberg L. J. Altabe M. Tantleef-Dunn S. (1999). “Theory assessment, and treatment of body image disturbance,” in Exacting Beauty: Theory, Assessment, and Treatment of body Image Disturbance, eds. Thomson J. K. Heinberg L. J. Altabe M. N. Tantlee-Dunn S. (Washington, DC: American Psychological Association).

[ref88] Tiggemann M. Miller J. (2010). The internet and adolescent girls’ weight satisfaction and drive for thinness. Sex Roles 63, 79–90. doi: 10.1007/s11199-010-9789-z

[ref89] Tiggemann M. Slater A. (2001). A test of objectification theory in former dancers and non-dancers. Psychol. Women Q. 25, 57–64. doi: 10.1111/1471-6402.00007

[ref90] Tiggemann M. Slater A. (2014). Nettweens: the internet and body image concerns in preteenage girls. J. Early Adolesc. 34, 606–620. doi: 10.1177/0272431613501083

[ref91] Valkenburg P. M. Peter J. (2013). The differential susceptibility to media effects model. J. Commun. 63, 221–243. doi: 10.1111/jcom.12024

[ref92] Varnes J. R. Stellefson M. L. Janelle C. M. Dorman S. M. Dodd V. Miller M. D. (2013). A systematic review of studies comparing body image concerns among female college athletes and non-athletes, 1997–2012. Body Image 10, 421–432. doi: 10.1016/j.bodyim.2013.06.001, 23856303

[ref93] Voelker D. K. Gould D. Reel J. J. (2014). Prevalence and correlates of disordered eating in female figure skaters. Psychol. Sport Exerc. 15, 696–704. doi: 10.1016/j.psychsport.2013.12.002

[ref94] Vogel E. A. Rose J. P. Roberts L. R. Eckles K. (2014). Social comparison, social media, and self-esteem. Psychol. Pop. Media Cult. 3:206. doi: 10.1037/ppm0000047

[ref95] Wells A. (2011). Metacognitive Therapy for Anxiety and depression. New York, NY: Guilford press.

[ref96] Wolf E. J. Harrington K. M. Clark S. L. Miller M. W. (2013). Sample size requirements for structural equation models: an evaluation of power, bias, and solution propriety. Educ. Psychol. Meas. 73, 913–934. doi: 10.1177/0013164413495237, 25705052 PMC4334479

[ref97] Zhu B. (1994). The revised Chinese norm of the competitive state anxiety inventory (Csai-2). J. Psychol. Sci. 17, 358–362,385.

