# Supplementary material for: A developmental perspective on appearance-related social media use, body dissatisfaction, and competitive anxiety among Chinese adolescent athletes: a three-wave longitudinal study
Source: Front Psychol. 2026 Apr 16;17:1805798. doi: 10.3389/fpsyg.2026.1805798 (PMC13128571; doi:10.3389/fpsyg.2026.1805798)
Supplement: Supplementary file 1 [file Table_1.docx]

**Supplementary Material**

**Table S1** Sensitivity Analysis: Decomposed ARSME Components Predicting Body Dissatisfaction (CLPM)

| **Sample** | **Path** | **β (Behavioral)** | **SE** | **p** | **β (Psychological)** | **SE** | **p** |
| --- | --- | --- | --- | --- | --- | --- | --- |
| Full sample (N = 356) | T1 → BD T2 | −.05 | .045 | .299 | .05 | .045 | .301 |
|  | T2 → BD T3 | −.06 | .044 | .152 | .08† | .044 | .066 |
| Younger (14–16, n = 200) | T1 → BD T2 | −.09 | .069 | .211 | −.08 | .069 | .234 |
|  | T2 → BD T3 | −.12† | .069 | .073 | −.07 | .069 | .323 |
| Older (17–18, n = 156) | T1 → BD T2 | −.09 | .079 | .246 | .03 | .078 | .693 |
|  | T2 → BD T3 | −.01 | .081 | .880 | .09 | .081 | .292 |

**Note.** All models control for prior body dissatisfaction, sport type, and prior competitive anxiety. Behavioral engagement = z-standardized daily social media time; Psychological investment = z-standardized Appearance-Related Social Media Consciousness (ASMC) score. Standardized coefficients (β) are reported. For age-stratified analyses, variables were additionally standardized within each subgroup. The reduced significance of individual components relative to the ARSME composite likely reflects attenuated statistical power when decomposing correlated sub-components into smaller subsamples. †p < .10. *p < .05. **p < .01. ***p < .001.
